# Medical expulsive therapy for ureter stone using naftopidil: A multicenter, randomized, double-blind, and placebo-controlled trial

**DOI:** 10.1371/journal.pone.0174962

**Published:** 2017-04-21

**Authors:** Sung Yong Cho, Woong Na, Sang Wook Lee, Min Chul Cho, Jong Jin Oh, Sangchul Lee, Juhyun Park, Soyeon Ahn, Chang Wook Jeong

**Affiliations:** 1 Department of Urology, Seoul Metropolitan Government- Seoul National University Boramae Medical Center, Seoul, Korea; 2 Department of Urology, National Medical Center, Seoul, Korea; 3 Department of Urology, Clinical Research Institute, Kangwon National University Hospital, Kangwon National University School of Medicine, Chunchon, Korea; 4 Department of Urology, Seoul National University Bundang Hospital, Seongnam, Korea; 5 Medical Research Collaboration Center, Seoul National University Bundang Hospital, Seongnam, Korea; 6 Department of Urology, Seoul National University Hospital, Seoul, Korea; Eberhard Karls University, GERMANY

## Abstract

**Objectives:**

A prospective, multicenter, randomized, double-blind, placebo-controlled trial evaluated the effects of naftopidil 75 mg for medical expulsive therapy for a single ureter stone.

**Materials and methods:**

Patients diagnosed with a ureter stone were prescribed aceclofenac 100 mg or a combined medication of tramadol 37.5 mg and acetaminophen 325 mg. Patients then randomly received either naftopidil 75 mg or placebo. Primary endpoint was the stone passage rate at 14 days after medication.

**Results:**

The 150 patients enrolled in 6 institutions randomly received either naftopidil (n = 75) or placebo (n = 75). The percentages of ureter stone passed spontaneously 14 days after medication was 60.9% in the naftopidil group and 53.3% in the placebo group. Stone-free rates and the total use of analgesics showed no significant differences between the two groups. Stone-free rates at 14 days after medication were decreased when maximal stone size was increased: 39.4% (≥ 5 mm), 15.5% (≥ 6 mm), and 7.0% (≥ 7 mm).

**Conclusions:**

The use of naftopidil 75 mg once daily was not effective in increasing spontaneous stone passage rates or reducing analgesic use. The maximal stone size < 6 mm and the follow-up for two weeks would be appropriate for applying medical expulsive therapy to patients with a single ureter stone.

## Introduction

Urolithiasis occurs in 5–10% of the world's population. It is one of the main reasons for visits to urologists [1,2]. The goal of stone treatment is to remove all stones with minimal complications. Active removal of ureter stones < 10 mm usually includes drug medication, medical expulsive therapy (MET), shock-wave lithotripsy (SWL), and ureteroscopic lithotripsy (URS) [3]. The MET has received a great deal of research attention over the past 10 years. Data from randomized controlled studies and a meta-analysis involving calcium channel blockers and alpha-adrenergic agents have shown its efficacy [4–6].

There are three subtypes of alpha-adrenergic receptors: 1A, 1B, and 1D. Alpha-1D receptors are most abundant in the human distal ureter. Tamsulosin is a drug used to improve urination in men with an enlarged prostate. Because tamsulosin has alpha-1A and -1D selectivity, it has been believed to be effective for MET. The selectivity of tamsulosin for alpha-1A is 3.3-times higher than for alpha-1D [7,8]. Naftopidil is another alpha-1D adrenergic receptor antagonist, with hhe highest documented selectivity for alpha-1D receptor to date. The selectivity of naftopidil for alpha-1D is 3.1-times higher than that for alpha-1A [9]. Thus, the adrenergic effect for the alpha-1D receptor appear greater for naftopidil than tamsulosin. This implies that naftopidil will display higher efficacy of MET than tamsulosin. However, this has not been studied using a well-designed, randomized, placebo-controlled study. Furthermore, no definite recommendations for MET have been formulated concerning the appropriate maximal stone sizes or follow-up periods for MET.

The authors performed a prospective, multicenter, randomized, double-blind, placebo-controlled study to clarify the effect of naftopidil for MET including the appropriate maximal stone size and follow-up periods.

## Patients and methods

### Subjects and study design

Patient dababases from the Seoul Metropolitan Government- Seoul National University Boramae Medical Center, National Medical Center, Kangwon National University Hospital, Seoul National University Bundang Hospital, Seoul National University Hospital were analyzed. The study was approved by the Institutional Review Board of each institution (approval number 16-2012-10). The use of naftopidil as ureter stone treatment was approved by the Ministry of Food and Drug Safety (approval number 12449, June 4, 2013). This study has been registered at www.clinicaltrials.gov (NCT01952314); because of the review process, the registration was completed after some patients were already enrolled in the study. The study protocol and the use of patients’ database for recruitment and follow-up were approved by IRB of each institution before patient recruitment. Registration of all ongoing and related trials for this drug/intervention was confirmed. All participants provide their written informed consent to participate in this study. Individual identifiers were removed and their data were anonymously analyzed.

This study included patients > 20 years of age with a single ureter stone. The maximal diameter of the stones was 3–10 mm. Exclusion criteria were: presence of multiple ureter stones, renal or hepatic dysfunction, febrile urinary tract infection, breastfeeding or pregnant women, solitary kidney, hypersensitivity to naftopidil, current use of alpha blockers, calcium channel blockers or corticosteroid within 4 weeks, moderate-to-severe cardiovascular or cerebrovascular disease, and significant active medical illness or genetic disorders.

The guideline of MET for a ureter stone is based on the European Association of Urology. When a patient was first diagnosed with a ureter stone in a computed tomography (CT) scan, monotherapy with aceclofenac 100 mg or combination treatment with tramadol 37.5 mg and acetaminophen 325 mg was prescribed. Patients then randomly received naftopidil 75 mg or placebo for the study period. The naftopidil and placebo were packaged and labelled identically. Randomization was carried out by the Medical Research Collaboration Center of Seoul National University Bundang Hospital using random permuted blocks of different sizes. The size of the next block was randomly chosen from the available block sizes. Randomization was stratified by each recruiting study site. One person packed the 14-day supplay of tablets for each patient. All study staff at all hospitals were blinded to treatment allocation and remained blind until the end of the trial. Unblinding request was only granted when there was medical emergencies and when requested by a physician.

For the sample size estimation, the probability of stone passage among the controls was 54.3% [4]. If the true probability of stone passage among naftopidil group was 80.5%, 65 cases per each group would be needed to reject the null hypothesis that the medication would have no additional effect for MET with probability 0.9. The type I error probability associated with this study of this null hypothesis was 0.05. Assuming the drop-out rate of patients of 10%, a sample size of 75 cases for each group was chosen.

### Clinical parameters

Medical and stone treatment histories were taken for all patients. X-ray of the kidney, ureter, and bladder, and low-dose non-contrast CT scans were performed before medication at visit 0. Patients were followed up at day 14 (visit 1), 28 (visit 2), 60 (visit 3), and 90 (visit 4) after initiation of medication to evaluate the appearance of the ureter stone. The primary endpoint was the stone passage rate at 14 days (visit 1). Follow-up low-dose CT scans were performed to define the presence of stones at the time of follow-up only if clinically indicated. Patients were prescribed with the medication during the entire study period. The amount of analgesic used was also evaluated.

### Statistical analyses

All parameters were represented as frequency and percentage or mean with standard deviation. Comparative results between two groups were analyzed using independent t-test or Mann-Whitney U test. Analysis of categorical variables was performed with Chi-square test or Fisher’s exact test. Uni- and multivariate regression analyses were performed to determined the odds ratio (OR) and 95% confidence interval (CI) to assess the significant predictors of stone passage rate. Statistical analyses were done using the intention-to-treat protocol set of patients who were followed-up at the visit 1. Statistical significance was considered at P < 0.05. Statistical analyses were performed using IBM SPSS Statistics version 21.0.

## Results

### Patients’ characteristics

**Fig 1:**
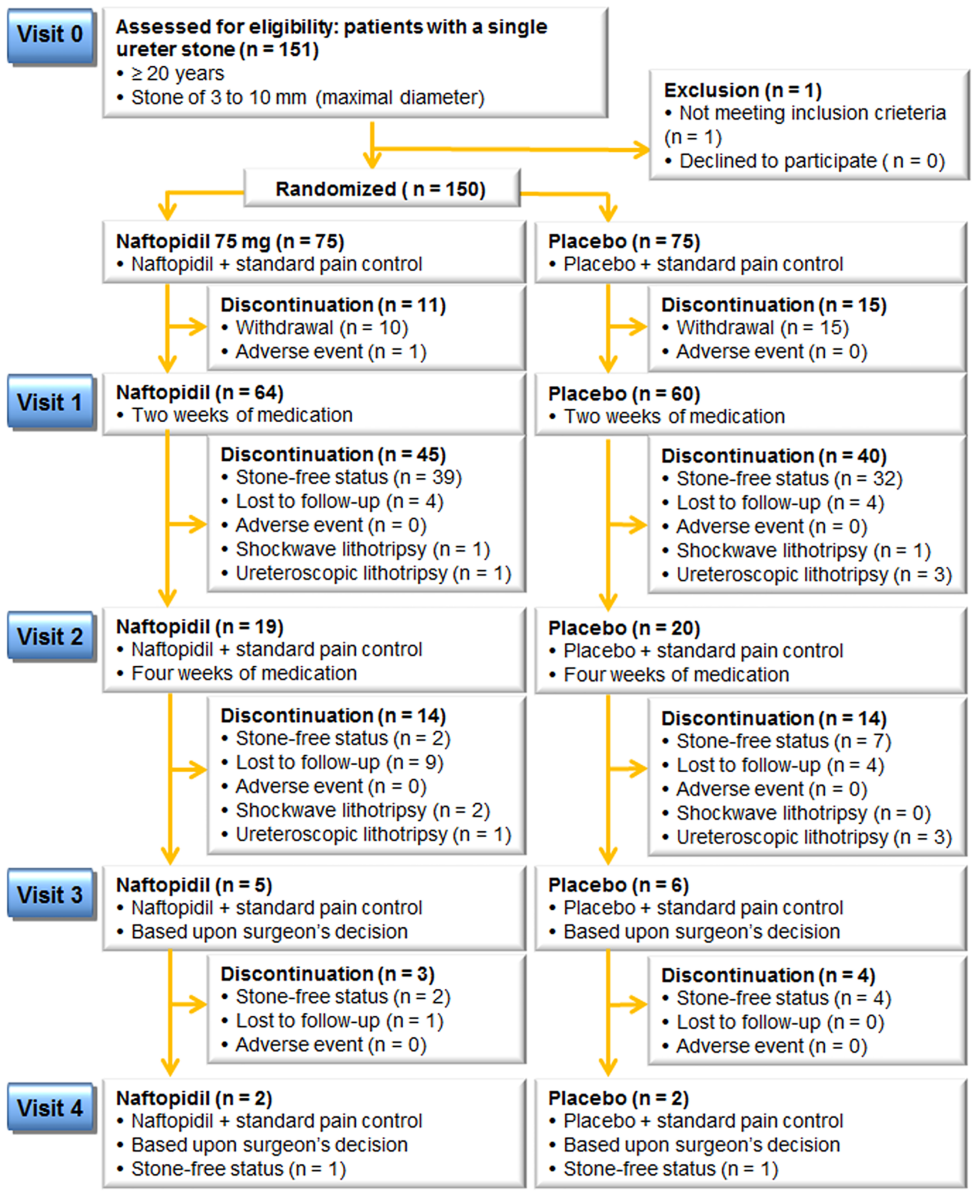
Flow diagram of the study.

The CONSORT diagram is shown in Fig 1. The 150 patients enrolled from six institutions were equally randomized to receive naftopidil 75 mg (n = 75) or placebo (n = 75). One patient was excluded due to the criteria violation. Sixty-four (85.3%) and 60 (80.0%) patients were followed-up after 2 weeks of medication. SWL was carried out for three patients in the naftopidil group and one patient in the placebo groups, respectively, while two and six cases of URS were performed for the naftopidil and placebo groups, respectively.

Patient demographics and stone characteristics are summarized in Table 1. There were no significant differences in mean age, sex ratio, comorbidity, renal function, or stone characteristics. Stones were usually located in the upper and lower ureter. Previous history of stone treatment did not differ significantly between the two groups (P = 0.176).

**Table 1:** Patients and stone characteristics (per protocol set at visit 1).

	Naftopidil 75mg	Placebo	P value
**No. of patients**	64	60	
**Patient characteristics**			
Mean age, years	48.1 ± 14.2	48.9 ± 13.8	0.748
Gender (M:F)	49:15	41:19	0.305
Mean BMI*, kg/m^2^	24.7 ± 2.8	24.8 ± 3.4	0.765
Comorbidities			
No. Diabetes mellitus, no (%)	3 (4.7)	4 (6.7)	0.633
No. Hypertension, no (%)	6 (9.4)	12 (20.0)	0.093
Renal function			
Creatinine, mg/dl	0.9 ± 0.20	0.9 ± 0.2	0.654
GFR*, mL/min/1.73m^2^	94.7 ± 25.6	114.8 ± 177.4	0.395
**Stone characteristics**			
Laterality (right:left)	34:30	35:25	0.560
Maximal stone size, mm	5.2 ± 1.4	4.8 ± 1.5	0.191
Stone volume, mm^3^	54.0 ± 44.1	52.2 ± 60.5	0.852
No. Radiolucent, no (%)	9 (14.1%)	4 (6.8%)	0.189
**Stone Location**			0.406
No. Upper ureter, no (%)	28 (43.8)	32 (53.3%)	
No. Midureter, no (%)	6 (9.4)	7 (11.7%)	
No. Lower ureter, no (%)	30 (46.9)	21 (35.0%)	
**Cumulative stone-free rates**			
No. Visit 1 (14 days), no (%)	39/64 (60.9)	32/60 (53.3)	0.392
No. Visit 2 (28 days), no (%)	41/64 (64.1)	39/60 (65.0)	0.717
No. Visit 3 (50 days), no (%)	43/64 (67.2)	42/60 (70.0)	0.682
No. Visit 4 (90 days), no (%)	44/64 (68.8)	43/60 (71.7)	0.987
Mean visits for stone-free status	1.2 ± 0.7	1.4 ± 0.7	0.359
**Total use of analgesics**	5.7 ± 10.5	3.6 ± 5.9	0.169
Use of aceclofenac	3.7 ± 7.0	2.8 ± 4.6	0.387
Use of acetaminophen and tramadol	2.0 ± 4.4	0.8 ± 2.2	0.062

BMI, body mass index; GFR, Glomerular Filtration Rate

### Primary and secondary endpoints according to the medication

More than half of the ureter stones were passed spontaneously at 14 days after medication in the naftopidil group (39 of 64, 60.9%) and the placebo group (32 of 60, 53.3%) (P = 0.468). Spontaneous stone passage rates continued to increase at each visit. However, the increasing portion of stone passage rates was small after two weeks of medication (from 60.9% to 68.8% in the naftopidil group, and from 53.3% to 71.7% in the placebo group). Stone-free rates showed no significant difference between the two groups. Spontaneous stone passage rates did not differ significantly according to stone location. Dizziness occurred in one patient in the naftopidil group at visit 1, and was not related to the presence of hypotension. Total use of analgesics showed no significant difference between the two groups. Stone migration rates did not show any significant differences either between the two groups (data not shown).

### Predictors for stone-free status

Uni- and multivariate logistic regression analyses revealed body mass index and maximal stone size as significant predictors for spontaneous stone passage rates at 14 days (Table 2). Use of naftopidil was not a significant predictor. Stone-free rates at 14 and 28 days decreased according to increasing maximal stone size: 39.4% and 40.0% (≥ 5 mm), 15.5% and 16.3% (≥ 6 mm), 7.0% and 6.3% (≥ 7 mm), and 4.2% and 3.8% (≥ 8 mm), respectively.

**Table 2:** Uni- and multivariate logistic regression analysis to predict stone-free status at 14 days after medication.

	Univariate			Multivariate		
	P value	OR	95% CI	P value	OR	95% CI
Age	0.521	1.012	0.975–1.051			
Gender (male versus female)	0.914	0.943	0.323–2.754			
Body mass index	0.049*	0.865	0.748–0.999	0.031*	0.855	0.741–0.986
Presence of diabetes	0.364	0.407	0.058–2.833			
Presence of hypertension	0.206	0.417	0.108–1.619			
Laterality (left versus right)	0.751	1.158	0.469–2.861			
Stone location	0.512	1.733	0.640–4.693			
Maximal stone size	0.002*	0.593	0.425–0.828	0.002*	0.616	0.451–0.842
Radiopacity	0.841	0.843	0.160–4.458			
Glomerular filtration rate	0.493	1.003	0.995–1.011			
Use of naftopidil	0.523	1.344	0.543–3.324			

### Subgroup analyses

Uni- and multivariate logistic regression analyses revealed differences between the naftopidil and placebo groups. In the naftopidil group, the maximal stone size (OR = 0.494, 95% CI 0.285–0.857, P = 0.012) was the only significant predictor. For stones with a maximal size < 5.5 mm, maximal stone size (OR = 0.040, 95% CI 0.001–0.829, P = 0.017) and glomerular filtration rate (GFR, OR = 1.088, 95% CI 1.011–1.170, P = 0.025) were significant predictors. In the placebo group, body mass index was the only significant predictor regardless of the maximal stone size (OR = 0.819, 95% CI 0.676–0.991, P = 0.040).

## Discussion

The effect of alpha-adrenergic agonists for MET remains debatable [10], with the effect of naftopidil on MET being unclear [11]. A well-designed placebo-controlled study that investigated the effect of naftopidil for MET reported a significantly improved time to stone expulsion [1]. This differs from the present study.

### Two weeks of medication before visit 1: High drop-out rates

At visit 1, only 85.3% and 80.0% of patients with a single ureter stone were followed up in the naftopidil and placebo groups, respectively. Only two of them were enrolled via the emergency department and a single case of dizziness occurred in the naftopidil group. The high drop-out rates might be the fact that patients suffered from great pain and they wanted to receive more active treatment such as SWL or URS than two weeks of medication. This number of drop-out rates would be similar to the rates of drop out in real clinical practice. Therefore, it is important for physicians to select appropriate patients for MET to increase their compliance rates for stone treatment.

### How long should we wait for MET?

The rate of spontaneous stone passage continued to increase, albeit marginally, with increasing number of clinic visits. Stone passage rates at 2 to 3 months were 7.9% in the naftopidil group and 18.4% in the placebo group. Therefore, 2 weeks of follow-up seems sufficient for MET. Otherwise, physicians should explain the possibility of an additional 10–20% of stone passage rates to patients when they recommend MET to their patients. These results are similar to the results of previous investigations [11,12] with mean or median stone passage time of about 8 days after medication.

### Ureteral stones > 6 mm did not pass well

Previous studies have shown an inverse relationship between the stone passage rates and stone size or location [13,14]. Presently, the stone-free rates also decreased according to increase in maximal stone size, from 39.4%–40.0% for stones ≥ 5 mm in size to 15.5–16.3% for stones ≥ 6 mm. Additionally, the stone passage rates were < 10% for stones ≥ 7 mm in size, indicating that stone passage rates were < 20% for cases with maximal stone size ≥ 6 mm. The findings indicate that it might not be appropriate for physicians to define a stone size of 6 mm as the definite cut-off level between MET and other active procedures. However, physicians should consider active treatments of SWL or URS after 2 weeks of medication in these cases.

### Predictors of maximal stone size and urine production

In the naftopidil group, the maximal stone size was the only significant predictor. We additionally sought to determine differences between smaller and larger stones. For smaller stones (i.e., < 5.5 mm), the glomerular filtration rate was also a significant predictor, indicating that increasing urine production according to high glomerular filtration rate would have advantages for MET with smaller stones than for larger stones. Further studies will be needed to confirm this suggestion.

### MET is really ineffective?

Previous investigations show the positive results of MET. Seitz C et al performed a pooled analysis which suggested that MET with alpha blockers or calcium channel blocker increased stone expulsion rate [4]. They analyzed 47 articles in which alpha blockers were tamsulosin, doxazosin, nifedipine, terazosin, and alfuzosin. No naftopidil was included. The authors mentioned that the majority of previous randomized studies which showed the positive effect of alpha blockers were small, single-center studies. Zhu Y et al reported a meta-analysis of seven trials about the role of tamsulosin to assist stone clearance after SWL [6]. However, the authors mentioned that the seven trials showed large heterogeneity between trials and a high quality confirmatory trial would be warranted. This would indicate the possibility of publication bias or selection bias.

### Limitations of this study

This study was limited the unanticipated high drop-out rates. However, this would not influence the effect of naftopidil on MET. Although the number of patients enrolled was small, the present results will be helpful to physicians who consider MET for patients with a ureter stone using naftopidil.

## Conclusions

The use of naftopidil 75 mg once daily was not effective in increasing spontaneous stone passage rate or reducing analgesic use. A maximal stone size < 6 mm and a follow-up of 2 weeks are appropriate for MET for patients with a single ureter stone.

## Supporting information

S1 Fig Clinical trial.gov_for MET. (PDF)

S1 CONSORT Checklist (CONSORT 2010 checklist-MET). (DOC)

S1 File Study design summary. (DOCX)

S2 File renamed_475ab (study protocol in Korean version). (DOCX)

S3 File renamed_475ab (Study protocol in English version). (DOCX)
