# Supplementary material for: Medical expulsive therapy for ureter stone using naftopidil: A multicenter, randomized, double-blind, and placebo-controlled trial
Source: PLoS One. 2017 Apr 21;12(4):e0174962. doi: 10.1371/journal.pone.0174962 (PMC5400235; doi:10.1371/journal.pone.0174962)
Supplement: S1 Fig — (PDF) [file pone.0174962.s001.pdf]

ClinicalTrials.gov
Protocol Registration System

Send message to ClinicalTrials.gov PRS
Help us improve: PRS Survey

Logout

Edit Protocol/Results Record

IMPORTANT: Chang Wook Jeong must Release this record.

Main Menu Select Preview Spelling Edit All **Problems** Delete Download XML

Title: Medical Explusive Therapy for Ureter Stone Using Naftopidil ID: SNUBH-URO-2012-03

Next Action: Release **Optional Actions: Reset to Completed Reset to In -Progress**

As Investigator and Responsible Party, **Chang Wook Jeong has sole authority to Release this record.**
About Responsible Party...

Record Status: Approved
In Progress | Completed | **Approved** | Released
Upload: Allowed [Set...]
Owned by: CWJeong Access List **Last updated:** 09/23/2013 01:54 by CWJeong
Initial release: [not yet released]

Add **Record Log:** None

**Edit**	**Unique Protocol ID:**	SNUBH-URO-2012-03
	**ClinicalTrials.gov ID:**	
	**Brief Title:**	Medical Explusive Therapy for Ureter Stone Using Naftopidil
	**Official Title:**	Medical Explusive Therapy for Ureter Stone Using Naftopidil: Multicenter, Randomized, Double-blind, Placebo Controlled Study
	**Study Type:**	Interventional
	**FDA Regulated Intervention?**	No
	**IND/IDE Protocol?**	No
**Edit**	**Secondary IDs:**	SNUBH-URO-2012-03-MET [SNU Bundang Hospital IRB]

For completed studies: [Record must have a ClinicalTrials.gov ID (NCT number) before results can be entered.]
About Results Data Entry
Delayed Results - Certification or Extension Request, per FDAAA

Edit **Responsible Party:** Principal Investigator
Investigator: Chang Wook Jeong [cwjeong]
Official Title: M.D, Ph.D.
Affiliation: Seoul National University Hospital

Sponsor: Seoul National University Hospital

Collaborators: Dong-A Pharmaceutical Co., Ltd.

Edit **Review Board:** Approval Status: Approved Approval Number: B-1210/175-007
Board Name: Seoul National University Bundang Hospital Institutional Review Board
Board Affiliation: Seoul National University Bundang Hospital Institutional Review Board
Phone: 82-31-787-1376 Email: snubhirb@gmail.com

Data Monitoring Committee? Yes

Oversight Authorities: Korea: Ministry of Food and Drug Safety

Edit **Brief Summary:**

This study is to investigate whether naftopidil is effective or not for the spontaneous passage of ureteral stones with sizes of 3 to 10 mm.

Detailed Description:

1. Enrollment
   a. patients with ureteral stones of sizes from 3 to 10 mm
   b. patients aged more than 18 years
2. Randomization
   a. naftopidil 75 mg qd for 14 days or plabebo
   b. Standard treatment with pain-killers were also applied.(aceclofenac)
3. Follow-up within 28 days
   a. We confirm the stone free status by CT or X-ray films at 14th and 28th days.
   b. Rates of active treatment will be also evaluated.

Edit **Record Verification Date:** September 2013

Overall Status: Not yet recruiting

Study Start Date: September 2013

Primary Completion Date: September 2014 [Anticipated]

Study Completion Date: September 2015 [Anticipated]

Edit **Study Design:** Primary Purpose: Treatment
Study Phase: Phase 3
Intervention Model: Parallel Assignment
Number of Arms: 2
Masking: Double Blind (Subject, Caregiver, Investigator, Outcomes Assessor)
Allocation: Randomized
Endpoint Classification: Safety/Efficacy Study
Enrollment: 150 [Anticipated]

Edit **Outcome Measures:** Primary Outcome Measure:

Title:	Stone passage rate at 14th day of medication
Time Frame:	14th day of medication
Description:	
Safety Issue?:	No

Secondary Outcome Measures:

Title:	stone passage rate at 28th day of medication
Time Frame:	28th day of medication
Description:	
Safety Issue?:	No

Title:	Day of stone passage within 4 weeks of medication
Time Frame:	at 28th day of medication
Description:	We will ask the day of stone passage during medication when we meet the patients at 14th and 28th day of medication.
Safety Issue?:	No

Title:	amount of analgesics used for 28 days of medication
Time Frame:	at 28th day of medication
Description:	We will ask the amount of medications when we meet the patients at 14th and 28th day of medication.
Safety Issue?:	No

Title:	Rate of active treatment

Time Frame: at 28th day of medication

Description: Active treatments include shock-wave lithotripsy or other surgical methods.

Safety Issue?: No

Other Pre-specified Outcome Measures:

Edit **Conditions:** Urinary Stones

Keywords:

Edit **Arms:** Placebo Comparator: Control groups with only analgesics

Control groups will receive only analgesics.

Active Comparator: Naftopidil

This interventional group will receive analgesics and naftopidil 75mg po qd.

Interventions: Drug: Naftopidil 75mg po qd

Other Names:

Flivas(TM) in South Korea

naftopidil 75mg po qd for 28 days with standard analgesic treatment

Edit **Eligibility Criteria:**

Inclusion Criteria:

- >= 18 years
- single 3 to 10 mm ureter stone (longest diameter)

Exclusion Criteria:

- Presence of multiple ureter stones
- Renal insufficiency (serum Cr > 1.4)
- Febile urinary tract infections
- pregnancy or breast feeding
- solitary kidney
- hypersensitivity to naftopidil
- current use of any alpha-blocker, calcium-channel blocker, corticosteroid (within 4 weeks)
- moderate or severe cardiovascular or cerebrovascular disease
- hepatic dysfunction (>2 x normal)
- significant active medical illness which in the opinion of the investigator would preclude protocol treatment

Gender: Both

Minimum Age: 18 Years

Maximum Age: 80 Years

Accepts Healthy Volunteers? No

Edit **Central Contact:** Chang Wook Jeong, M.D., Ph.D.
Telephone: 82-10-8933-4353
Email: drboss@korea.com

Central Contact Backup: Sung Yong Cho, M.D., Ph.D.
Telephone: 82-10-6287-0217
Email: kmoretry@daum.net

Edit **Study Officials/Investigators:** Chang Wook Jeong, M.D., Ph.D.
Study Principal Investigator
Seoul National University Hospital

Edit **Locations:** **Facility:** Seoul National University Bundang Hospital
Seongnam, Kyunggi, Korea, Republic of 463-712
Contact: Sangchul Lee, M.D., Ph.D.
Investigator: Sangchul Lee, M.D
Role: Principal Investigator
Recruitment Status: Not yet recruiting

Facility: CHA Bundang Medical Center, Bundang hospital
Seongnam, Kyunggi, Korea, Republic of 463-712
Contact: Jong Jin Oh, M.D
Investigator: Jong Jin Oh, M.D
Role: Principal Investigator
Recruitment Status: Not yet recruiting

Facility: Seoul National University Boramae Medical Center
Seoul, Korea, Republic of
Contact: Sung Yong Cho, M.D., Ph.D.
Email: kmoretry@daum.net
Investigator: Sung Yong Cho, M.D., Ph.D.
Role: Principal Investigator
Recruitment Status: Not yet recruiting

Facility: Donguk University Ilsan Hospital
Goyang, Kyunggi, Korea, Republic of
Contact: Minchul Cho, M.D., Ph.D.
Investigator: Minchul Cho, M.D., Ph.D.
Role: Principal Investigator
Recruitment Status: Not yet recruiting

Facility: Kangwon National University Hospital
Chuncheon, Korea, Republic of
Contact: Sang Wook Lee, M.D., Ph.D.
Investigator: Sang Wook Lee, M.D., Ph.D.
Role: Principal Investigator
Recruitment Status: Not yet recruiting

Facility: National Medical Center
Seoul, Korea, Republic of
Contact: Woong Na, M.D., Ph.D.
Investigator: Woong Na, M.D., Ph.D.
Role: Principal Investigator
Recruitment Status: Not yet recruiting

Edit **Citations:**

Edit **Links:**
