# Supplementary material for: Medical expulsive therapy for ureter stone using naftopidil: A multicenter, randomized, double-blind, and placebo-controlled trial
Source: PLoS One. 2017 Apr 21;12(4):e0174962. doi: 10.1371/journal.pone.0174962 (PMC5400235; doi:10.1371/journal.pone.0174962)
Supplement: S1 File — (DOCX) [file pone.0174962.s003.docx]

**Summary of IRB documents**

**[Title]**

Medical Expulsive Therapy (MET) for ureter stone by using Naftopidil: Multicenter, randomized, double-blind, placebo controlled study.

**[Responsible Party]**

Principal Investigator: Chang Wook Jeong [cwjeong] M.D, Ph.D.

Affiliation: Seoul National University Hospital

Sponsor: Seoul National University Hospital

Collaborators: Dong-A Pharmaceutical Co., Ltd.

**[Investigators]**

Sung Yong Cho, Juhyun Park, Min Chul Cho (Department of Urology, Seoul Metropolitan Government- Seoul National University Boramae Medical Center, Seoul, Korea)

Woong Na (Department of Urology, National Medical Center, Seoul, Korea)

Sang Wook Lee (Department of Urology, Clinical Research Institute, Kangwon National University Hospital, Kangwon National University School of Medicine, Chunchon, Korea)

Jong Jin Oh, Sangchul Lee (Department of Urology, Seoul National University Bundang Hospital, Seongnam, Korea)

Soyeon Ahn (Medical Research Collaboration Center, Seoul National University Bundang Hospital, Seongnam, Korea)

Chang Wook Jeong (Department of Urology, Seoul National University Hospital, Seoul, Korea)

**[Edit Review Board]**

Approval Number: B-1210/175-007

Board Name: Seoul National University Bundang Hospital, Institutional Review Board

Phone: 82-31-787-1376 Email: snubhirb@gmail.com

Oversight Authorities: Korea: Ministry of Food and Drug Safety

**[Objective**]

This study is to investigate whether naftopidil is effective or not for the spontaneous passage of ureteral stones with sizes of 3 to 10 mm.

**[Introduction]**

Urolithiasis occurs in 5-10% of the world's population, which is one of the most common reasons to visit urology department.

Over the past 10 years, MET has been considered as an effective way to treat Urolithiasis. Especially, alpha-adrenergic agents have shown the efficacy as a MET. Alpha-adrenergic receptors have three different subtypes which are 1A, 1B, and 1D. Among three subtypes, Alpha-1D receptors are found most commonly in the human distal ureters.

Previous studies have shown that the alpha 1A selectivity of tamsulosin is 3.3 times higher than that for alpha 1D, meanwhile alpha 1D sensitivity of naftopidil is 3.1 times higher than that for alpha 1A. However, there is no sufficient data to prove efficacy of naftopidil for MET and no definite recommendation such as appropriate maximal stone sizes and follow-up periods. In order to demonstrate these topics for MET, a well-designed prospective study is mandatory.

Therefore, the authors performed a prospective, multicenter, randomized, double-blind, placebo-controlled study to investigate the effect of naftopidil, the appropriate maximal stone size and follow-up periods for MET.

**[Patients and Methods]**

- Materials and methods

When the patients were diagnosed with a ureter stone, the aceclofenac 100 mg or the combined medication of tramadol 37.5 mg and acetaminophen 325 mg was prescribed first. Then the patients were 1:1 randomized to receive either naftopidil 75 mg or placebo. Primary endpoint was the stone passage rates at 14 days after medication.

- Enrollment

<Inclusion criteria>

a. ≥ 20 years

b. Patients with a single 3 to 10 mm ureter stone (longest diameter)

<Exclusion criteria>

a. Presence of multiple ureter stones

b. Renal insufficiency (serum Cr > 1.4)

c. Febrile UTI (fever > 38°, evidence of urinary infection)

d. Pregnancy or breast feeding

e. Solitary kidney

f. Hypersensitivity to Naftopidil

g. Current use of any alpha-blocker, calcium-channel blocker, corticosteroid (within 4 weeks)

h. Moderate or severe cardiovascular or cerebrovascular disease

i. Hepatic dysfunction (>2 x normal LFT)

j. Significant active medical illness which in the opinion of the investigator would preclude protocol treatment

h. Genetic disorder such as Galatose intolerance, Lapp Lactase deficiency, Glucose-Galactose malabsorption

- Sample size estimation

We are planning a study of independent cases and controls with 1 control(s) per case. Prior data indicate that the probability of exposure among controls is 0.543. (RR 1.45 [1.34-1.57])(Seitz C et al: "Medical Therapy to facilitate the passage of stones: What is the evidence?" Eur Urol 2009;56:455-71)) If the true probability of exposure among cases is 0.805, we will need to study 65 case patients and 65 control patients to be able to reject the null hypothesis that the exposure rates for case and controls are equal with probability (power) 0.9. The Type I error probability associated with this test of this null hypothesis is 0.05. We will use an uncorrected chi-squared statistic to evaluate this null hypothesis. The drop-out rate was 10%. Finally the number of patients included should be 150 for two groups.

- Study design

- Randomization

a. Randomization would be carried out by the Medical Research Collaboration Center of Seoul National University Bundang Hospital

b. naftopidil 75 mg qd for 14 days or plabebo (1:1)

c. Randomization would be stratified by each recruiting study site and permuted-block random allocation with varying block sizes would be used.

d. Standard treatment with pain-killers were also applied.(aceclofenac). A single person will pack the 14 days’ tablets of each patient.

e. double-blinded to patients and investigators

- Follow-up within 28 days.

a. We confirm the stone free status by CT or X-ray films at 14th and 28th days.

b. Rates of active treatment will be also evaluated.

**[Study design]**

Primary Purpose: Treatment

Study Phase: Phase 3

Intervention Model: Parallel Assignment

Number of Arms: 2

Masking: Double Blind (Subject, Caregiver, Investigator, Outcomes Assessor)

Allocation: Randomized

Endpoint Classification: Safety/Efficacy Study

Enrollment: 150 [Anticipated]

**[Outcome Measures]**

- Primary Outcome Measure: Stone passage rate at 14th day of medication
- Secondary Outcome Measures:

a. stone passage rate at 28th day of medication

b. Day of stone passage within 4 weeks of medication

c. amount of analgesics used for 28 days of medication

**[Time Table]**

| Contents | Procedure (months) | | | | | | | | | | | | | 비고 | |
| --- | --- | --- | --- | --- | --- | --- | --- | --- | --- | --- | --- | --- | --- | --- | --- |
|  | 1 | 2 | 3 | 4 | 5 | 6 | 7 | 8 | 9 | 10 | 11 | 12 |  | |  |
| Randomization |  |  |  |  |  |  |  |  |  |  |  |  |  | |  |
| Enrollment |  |  |  |  |  |  |  |  |  |  |  |  |  | |  |
| F/u periods |  |  |  |  |  |  |  |  |  |  |  |  |  | |  |
| Data collection |  |  |  |  |  |  |  |  |  |  |  |  |  | |  |
| Data analysis |  |  |  |  |  |  |  |  |  |  |  |  |  | |  |
| Submission of articles |  |  |  |  |  |  |  |  |  |  |  |  |  | |  |
