# Supplementary material for: Medical expulsive therapy for ureter stone using naftopidil: A multicenter, randomized, double-blind, and placebo-controlled trial
Source: PLoS One. 2017 Apr 21;12(4):e0174962. doi: 10.1371/journal.pone.0174962 (PMC5400235; doi:10.1371/journal.pone.0174962)
Supplement: S2 File — (DOCX) [file pone.0174962.s004.docx]

**연구계획서**

나프토피딜을 이용한 요관 결석에 대한 내과적 배출 요법: 다기관, 무작위배정, 양쪽눈가림, 위약 대조군 연구

(SNUBH-URO-2012-03)

Ver. 2.0

서울대학교 의과대학 / 서울대학교병원

비뇨기과 정 창 욱 교수

**연구개요**

| 연구제목 | (국문) 나프토피딜을 이용한 요관 결석에 대한 내과적 배출 요법: 다기관, 무작위배정, 양쪽눈가림, 위약 대조군 연구 |
| --- | --- |
|  | (영문) Medical expulsive therapy for ureter stone using Naftopidil: Multicenter, randomized, double-blind, placebo controlled study. |
| 책임연구자 | 비뇨기과 정창욱 교수 |
| 연구비 지원기관 | 없음 |

| 연구 목적 | Alpha-1D selective adrenergic blocker인 나프토피딜이 요관 결석에 대한 배출 촉진 효과가 있는지 확인하기 위한 연구임 |
| --- | --- |
| 연구 설계 | 전향적, 다기관, 무작위배정, 양쪽눈가림, 위약 대조군 연구. |
| 연구 기간 | IRB승인일 ~ 2014년 11월 16일 |
| 연구 대상  (시험약 등) | 성인 요관결석 환자  주요 선정기준>  1. 만 20세 이상  2. 3-10mm 크기의 단일 요관 결석 환자 (장경 기준) |
| 연구 대상자 수 | 전체 150명, 경쟁적 모집으로 본기관 대상자수 미정 |
| 취약한 연구대상자 | 없음 |
| 연구 방법 | 시험약으로 naftopidil 75mg p.o. qd로 사용하며 위약을 대조약으로 한다. 14일간의 투약과 추적 관찰을 통해 양군간의 결석 배출률을 비교한다. 전체 추적 관찰은 28일 간 시행한다. 연구의 기본적인 개요는 아래 다이어그램과 같다.   |
| 유효성 평가 | 2주째 요관 결석 배출률 |
| 안전성 평가 | 이상반응 발생시 이상 반응 내용 및 중대성 여부 (사망, 생명에 위협, 입원, 장애/불구, 선천성 기형/이상 초래, 중요한 의학적 사건, 기타), 예상 여부 (예, 아니오), 인과관계 (확실함, 거의확실함, 가능함, 가능성적음, 평가곤란, 평가불가, 관련없음)를 평가 |
| 기대효과 및  예상결과 | 요관결석 환자에서 Naftopidil 75mg 복용이 위약에 비해 결석 배출률을 높이고, 배출 시간을 단축 시켜줄 것으로 예상됨. |

1. **연구의 명칭 및 단계**

(국문) 나프토피딜을 이용한 요관 결석에 대한 내과적 배출 요법: 다기관, 무작위배정, 양쪽눈가림, 위약 대조군 연구.

(영문) Medical expulsive therapy for ureter stone using Naftopidil: Multicenter, randomized, double-blind, placebo controlled study.

1. **연구의 실시기관 명칭 및 주소**

(실시 기관명) 서울대학교병원 비뇨기과

(주소) 110-744, 서울특별시 종로구 대학로 101

1. **연구책임자 및 담당자**
2. **연구책임자**

연구책임자: (성명) 정창욱 (직명) 서울대학교병원 비뇨기과 조교수

1. **공동연구자**

총괄연구책임자: (성명) 정창욱 (직명) 서울대학교병원 비뇨기과 조교수

**원내 공동 연구자>**

공동 연구자: (성명) 김명 (직명) 서울대학교병원 비뇨기과 전임의

공동 연구자: (성명) 이영주 (직명) 서울대학교병원 비뇨기과 전임의

**타기관 공동 연구자>**

공동 연구자: (성명) 정현 (직명) 보라매병원 비뇨기과 부교수

공동 연구자: (성명) 이승배 (직명) 보라매병원 비뇨기과 조교수

공동 연구자: (성명) 조성용 (직명) 보라매병원 비뇨기과 진료교수

공동 연구자: (성명) 조민철 (직명) 일산동국대학교병원 비뇨기과 조교수

공동 연구자: (성명) 배정범 (직명) 일산동국대학교병원 비뇨기과 진료교수

공동 연구자: (성명) 이상욱 (직명) 강원대학교병원 비뇨기과 부교수

공동 연구자: (성명) 박홍주 (직명) 강원대학교병원 비뇨기과 조교수

공동 연구자: (성명) 이종복 (직명) 국립중앙의료원 비뇨기과 과장

공동 연구자: (성명) 나웅 (직명) 국립중앙의료원 비뇨기과 진료의

공동 연구자: (성명) 이상철 (직명) 분당서울대학교병원 비뇨기과 조교수

공동 연구자: (성명) 오종진 (직명) 분당서울대학교병원 비뇨기과 조교수

1. **연구담당자**

공동 연구자: (성명) 김명 (직명) 서울대학교병원 비뇨기과 전임의

1. **임상시험용 의약품 관리약사 /임상시험용 의료기기 관리자 성명 및 직명**

**의생명연구원 중앙임상시험센터 약국**

장홍원 (직명) 임상시험 약무파트장

박향미 (직명) 관리약사

김성환 (직명) 관리약사

이진아 (직명) 관리약사

김잔디 (직명) 관리약사

김민경 (직명) 관리약사

1. **연구 의뢰자명(연구비 지원기관) 및 주소(모니터 직명 및 성명 포함)**

시험자 주도 임상 시험이며, 연구비 지원기관 없음.

1. **연구의 목적 및 배경**

**1) 연구배경 및 필요성**

요로 결석은 전세계 인구의 5-10%에서 발생하고 가장 흔하게 비뇨기과를 찾는 이유 중에 하나이다. 또한 여러 연구에서 세계적으로뿐만 아니라 한국에서도 그 유병률이 증가하는 추세이다. 이러 요관 결석 치료의 목표는 합병증을 최소화하면서 완전히 결석을 제거하는 것이다. 이를 위해 결석의 크기와 위치를 고려하여 대기요법, 약물요법, 체외충격파쇄석술 (SWL), 요관경하요관절석술 등이 표준적으로 사용되고 있다. 특히 약물을 이용한 내과적 배출요법 (medical expulsive therapy: MET)가 지난 10여 년간 크게 주목을 받으며 연구들이 수행되었다. 여러 약물들 중에 calcium channel blocker와 alpha-adrenergic blocker가 무작위 배정 연구들을 통해 그 효과가 입증되었다. 특히 tamsulosin을 위시한 alpha-blocker에 대한 증거들이 축적되어 무작위 배정 연구들에 대한 메타분석을 통해서도 명확히 효과가 입증되어 MET에 주로 사용되고 있다. 한국인에 있어서도 tamsulosin이 요관 결석의 MET에 효과적이라는 것이 본 연구진에 의해서도 메타분석을 통해 증명된바 있다 (Korean Journal of Urology 게재 예정).

Alpha-adrenergic receptor에는 1A, 1B, 1D의 세가지 subtype이 있으며, 사람의 요관에는 apha-1D receptor가 가장 풍부하게 존재하며, 특히 하부 요관에 밀도가 가장 높은 것으로 알려져 있다. Tamsulosin이 비교적 다른 alpha-blocker에 비해 효과적이라고 여겨지는 것은 tamsulosin이 alpha-1A 및 -1D에 선택적이며 기존의 다른 alpha-blocker에 비해 1D에 대한 선택성이 높기 때문으로 추정되었다.

Naftopidil은 국내에는 최근 출시된 alpha-blocker로 지금까지 나온 약물 중 가장 alpha-1D에 선택성이 높은것으로 알려져 있다. Tamsulosin은 alpha-1A에 대한 선택성이 alpha-1D에 비해 3.3배 높지만, naftopidil은 오히려 alpha-1D에 대한 선택성이 alpha-1A에 비해 3.1배 높다. 따라서, naftopidil이 tamsulosin에 비해 alpha-1D에 대한 길항 효과는 약 9배 가장 높다고 볼 수 있다. 따라서, 이론적으로 naftopidil은 요관 결석의 MET에 매우 효과적일 것으로 추정된다. 하지만 아직까지 이에 대한 잘 디자인된 전향적 연구가 없는 실정이다. 기존에 이와 관련된 두 연구가 있었다. 한 연구는 다른 alpha blocker인 silodosin과의 open label 비교 연구였고, 한 연구는 위약 없는 open label 연구였다. 또한 이 연구에서는 요관 결석 배출률이 90.9% vs 26.7% (p<0.001)로 대조군에서 일반적으로 알려진 것보다 너무 낮게 나와 연구의 신뢰성에 의문이 제기된다.

따라서, 요관 결석에 대한 MET에 대한 naftopidil의 효과를 명확히 증명하기 위해서는 잘 디자인된 전향적 연구가 절실히 필요한 상황이다. 이에 본 연구자들은 전향적, 다기관, 무작위배정, 양쪽눈가림, 위약 대조군 연구를 통해 이를 확인하고자 한다.

이 연구를 통하여 naftopidil의 요관 결석에 대한 배출 효과가 확인된다면 향후 임상에 적용하여 많은 환자들에 요관 배출을 빠르게 도와줘서 유병 기간을 줄여주고, 불필요한 적극적 치료를 회피할 수 있게 하여 합병증에 따른 고통을 피하고 사회적비용을 많이 절감할 수 있을 것으로 기대된다. 또한 이를 바탕으로 향후 naftopidil과 다른 alpha-blocker 특히 tamsulosin과의 직접 비교를 위한 이론적 근거가 마련되며, naftopidil과 다른 약제의 병용 요법이나 다양한 상황에서 MET의 효과를 확인해볼 이론적 근거가 마련될 것으로 기대됨.

**2) 연구목적**

본 연구에서는 성인에서 3-10mm 크기의 단일 요관 결석에 대한 치료로 naftopidil 75mg qd 14일 요법의 요관 결석 배출 효과를 확인하는 것이 목적임.

요관 결석의 크기를 3mm 이상으로 잡은 것은 그 이하로는 자연 배출이 매우 많을 것으로 판단되어 불필요할 가능성이 있고 10mm까지로 잡은 것은 closed observation 또는 MET가 사용될 수 있는 가이드 라인에 제시된 최대 크기임. (AUA/EAU guideline)

Primary outcome: 투약 2주째 요관 결석 배출률

Secondary outcome: 1. 4주째 요관 결석 배출률

2. 요관 결석 배출 기간 (4주 이내)

3. 진통제 요구량 (4주 이내)

4. 적극적 치료의 비율 (4주 이내)

1. **임상시험용 의약품 및 의료기기 코드명(또는 주성분의 일반명), 원료약품의 분량, 제형 등(대조약 포함)**

원내코드 NFTP75, Naftopidil 75mg, Tab. P.O. qd hs

임상시험센터 약품코드 YURONTP3, URO-2012-03용 Naftopidil 75mg/위약

1. **연구 대상 질환**

요관 결석

1. **연구대상자의 선정 기준, 제외기준, 목표한 대상자 수 및 산출 근거**

**모집방법:** 응급실 및 외래 방문 환자 중 영상 검사로 확진 되고 상기 선정 기준에 맞는 경우 본 임상 시험을 설명하고 동의하는 환자에 대해서만 참여시킴. 별도의 모집 광고 등은 시행하지 않음.

1. **선정기준**
   1. ≥ 20 years
   2. Patients with a single 3 to 10 mm ureter stone (longest diameter)
2. **제외기준**
   1. Presence of multiple ureter stones
   2. Renal insufficiency (serum Cr > 1.4)
   3. Febrile UTI (fever > 38°, evidence of urinary infection)
   4. Pregnancy or breast feeding
   5. Solitary kidney
   6. Hypersensitivity to Naftopidil
   7. Current use of any alpha-blocker, calcium-channel blocker, corticosteroid (within 4 weeks)
   8. Moderate or severe cardiovascular or cerebrovascular disease
   9. Hepatic dysfunction (>2 x normal LFT)
   10. Significant active medical illness which in the opinion of the investigator would preclude protocol treatment
   11. Genetic disorder such as Galatose intolerance, Lapp Lactase deficiency, Glucose-Galactose malabsorption
3. **목표한 대상자 수 및 산출 근거**
   1. 연구 대상수: 총 150명
   2. 대상 수 산출 근거: Naftopidild의 결석 배출률이 최소한 기존의 alpha-blocker 들과 같거나 우수하다고 가정하였다. 기존 Meta-analysis를 통해 알려진 alpha-blocker의 요관 결석 배출률은 80.5%, 위약의 배출률은 54.3%였다 (RR 1.45 [1.34-1.57])(Seitz C et al: "Medical Therapy to facilitate the passage of stones: What is the evidence?" Eur Urol 2009;56:455-71). 이를 바탕으로 alpha 0.05, power 90%, 배정비율 1:1을 기준으로 계산하면 각 군당 65명이 필요하며, 10% 가량의 drop-out을 고려하여 75명씩, 총 150명을 피험자의 수로 계산하였다.
4. **예상연구기간**

IRB 승인일부터 1년 (2014년 11월 16일)

1. **연구방법**
2. **연구방법개요**

전향적 다기관, 무작위배정, 양쪽눈가림, 위약 대조군 연구로 20세 이상 성인에서 3-10mm 크기의 단일 요관 결석을 진단받은 경우를 대상으로 한다. 시험약으로 naftopidil 75mg p.o. qd로 사용하며 위약을 대조약으로 한다. 14일간의 투약과 추적 관찰을 통해 양군간의 결석 배출률을 비교한다. 전체 추적 관찰은 28일 간 시행한다. 연구의 기본적인 개요는 아래 다이어그램과 같다.

본 연구는 순수 연구자 주도의 다기관 학술 연구로 경제적 이해관계로부터 완전히 독립적인 연구를 수행하기 위해 제약회사를 비롯한 타 기관의 금전적 지원을 일체 받지 않는다. 다만 연구 수행에 필수적으로 필요한 해당 약물과 위약은 해당 제약회사인 동아제약으로부터 조건 없이 제공 받기로 하였다. 또한 본 연구의 protocol은 약물의 복용이 추가될 뿐 현재 일반적으로 실제 임상에서 시행하는 검사와 추적 관찰과 동일하여 추가 관찰비용이 발생하지 않습니다. 또한 일반적으로 발생하는 연구비에 대해서는 각 기관에서 자비로 처리하기로 하였습니다.

또한 본 연구는 Naftopidil의 식약처 허가 적응증인 전립선비대증외의 환자에 사용하는 것으로 식약처 승인을 받았습니다 (제 12449호, 2013년 6월 4일).

1. **연구수행일정표**

IRB 승인 유효기간이 1년이므로 연장 필요 시 승인만료일 1개월 전에는 연차 지속심의를 받을 것임. (총 연구기간: 12개월)

| 연 구 내 용 | 추 진 일 정 (개월째) | | | | | | | | | | | | | 비고 | |
| --- | --- | --- | --- | --- | --- | --- | --- | --- | --- | --- | --- | --- | --- | --- | --- |
|  | 1 | 2 | 3 | 4 | 5 | 6 | 7 | 8 | 9 | 10 | 11 | 12 |  | |  |
| 난수표 작성 및 투약 준비 |  |  |  |  |  |  |  |  |  |  |  |  |  | |  |
| 환자 모집 및 투약 |  |  |  |  |  |  |  |  |  |  |  |  |  | |  |
| 추가 추적 관찰 |  |  |  |  |  |  |  |  |  |  |  |  |  | |  |
| 자료정리 |  |  |  |  |  |  |  |  |  |  |  |  |  | |  |
| 통계분석 |  |  |  |  |  |  |  |  |  |  |  |  |  | |  |
| 논문작성 및 투고 |  |  |  |  |  |  |  |  |  |  |  |  |  | |  |

1. **비교군 설정 및 무작위 배정 방법**

1. 비교군 설정: 위약

2. 무작위 배정:

-무작위 배정은 피험자와 직접 접촉하지 않는 독립적인 데이터 관리자 (분당서울대학교병원 의학연구협력센터: MRCC) 가 시행

-치료군과 대조군의 배정 비율 = 1:1

-기관별로 층화하여 각 기관별로 무작위 배정을 시행하고 별도의 무작위 배정표를 작성

-데이터 관리자는 무작위 배정 소프트웨어를 이용하여 일련번호에 permuted-block random allocation with varying block sizes 을 시행하고 리스트를 보관.

3. 눈가림법:

- 이중눈가림법

- 이 무작위 배정 결과는 나머지 연구자 및 환자, 보호자에게 철저히 비밀로 유지되며 최종 분석을 시행할 때 나머지 연구자에게 공개를 원칙으로 함.

- 약을 제공하는 제약회사 (동아 ST) 는 일련번호가 붙은 약 봉투에 배정 결과에 적합하게 시험약 또는 위약을 14일분 (14 tab.) 넣어 밀봉하고, 나머지 연구자들에게 약의 종류를 모르게 한 채 제공함. 해당 제약회사에서도 약의 밀봉을 담당한 1인에게만 random table을 공개하고, 회사의 임상 시험 담당자가 나머지 인원에게는 공개되지 않게 보관함. 이 random table은 최종 분석 이후 또는 피험자에게 영향을 미칠 수 있는 응급 상황에서만 공개하도록 함.

- 총괄 책임 연구자는 모든 기관의 random table을, 각 기관별 책임 연구자, 각 기관별 임상약국의 관리 약사는 해당 기관의 random table을 다른 사람에게 공개되지 않게 보관하며, 이 random table은 최종 분석 이후 또는 피험자에게 영향을 미칠 수 있는 응급 상황에서만 공개하도록 함.

1. **시험약 투여∙사용량, 투여∙사용 방법, 병용 요법, 대조약 사용시 그 선택사유**

1. 시험약 투여, 사용량, 사용방법: Naftopidil의 경우 국내에서 25, 50, 75mg Tab. 제형으로 공급되며 이 용량으로 전립선 비대증을 적응증으로 식약청 허가를 받았음. Naftopidil의 경우 100mg 미만에서는 25mg (2.6%), 50mg (2.0%), 75mg (1.2%) 등 용량 차이에 따른 부작용의 발현 빈도가 차이 나지 않음 (별첨 Flivas 신약신청자료집, P 224). 또한 양물에 대한 효과는 약물의 용량과 선형관계를 보이고 있음.

실제 임상에서의 경험 역시 약물 용량에 따른 부작용의 빈도나 정도가 차이 나지 않고, 다른 알파차단제의 일반적인 용량 (tamsulosin 0.2mg, doxazosin 4mg 등)과의 Naftopidil동등 용량이 75mg으로 인식되어 있음. 따라서, 적응증을 받은 전립선비대증 환자에 있어서도 75mg이 현실적인 표준 용량으로 사용되고 있으며, 특별히 dose escalation을 시행하지 않고 있음. 참고로 본 분당서울대학교병원에서 2012년 10월 한달 처방된 용량별 처방 건수를 확인하면 25mg 1건, 50mg 28건, 75mg 161건으로 이런 현실을 반영하고 있음.

만약 50mg 또는 25mg으로 용량으로 설정하였다가 충분한 efficacy를 보이지 못한다면, 75mg으로 추가 연구를 수행하여야 하고, 25, 50, 75mg을 모두 포함한 study design을 한다면 대상 환자가 급격히 늘어나게 됩니다. 또한 해당 약물 요법의 기간과 primary outcome의 time duration이 2주간으로 현실적으로 1-2주 간격으로 dose escalation이 현실적으로 어렵습니다.

따라서, 본 연구의 경우 Naftopidil이 medical expulsive therapy에 적용되는 well-designed RCT로는 첫 연구로 75mg qd 용법은 성인에서 안전하게 사용하면서 혈장 농도를 최대로 하여 그 효과를 가장 확실하게 알 수 있어 75mg을 시험 용량으로 설정하였습니다. 2-4주간의 경과 관찰 이후에는 보다 적극적인 치료를 시행하는 것이 신기능을 보호하고 여러 합병증을 줄이는 일반적인 치료 방법임. 따라서 Naftopidil 75mg qd 14일 요법을 선택함.

2. 대조약 사용시 그 선택사유: 위약 – 아직 요관결석의 배출요법으로 naftopidil의 효과가 위약 대조군 연구로 증명된 바 없음. 따라서, 위약을 대조약으로 선택하기로 함. 위약의 경우 naftopidil 75mg과 크기, 색상, 무게 등 성상이 동일하게 제조될 예정이며, 주성분을 제외한 첨가제의 종류 및 분량은 동일할 예정입니다. 구체적인 성분에 대해서는 실제 위약 생산 후 제공 시 성분 명세서 및 관련 자료는 같이 제공 받기로 되어 있습니다.

3. 병용요법: 일반적인 요관 결석 환자의 통증 조절 치료인 aceclofenac 100mg 또는 이로써 통증 조절이 되지 않을 때 Ultracet (tramadol 37.5mg/acetaminophen 325mg)을 필요에 따라 복용하는 것을 두 군 모두 허용함. 이로써 환자들이 기본적인 표준 치료를 받을 수 있게 하고 불필요한 통증을 느끼지 않게 보장함.

1. **관찰항목, 임상검사항목 및 관찰검사방법**

나이, 성별, 요관 결석의 좌우 위치, 첫 진단 시 결석의 위치 (상부, 중부, 하부), 결석의 최장 직경 (CT를 시행한 경우 CT를 우선으로 기준하며, KUB 또는 IVP만 시행한 경우는 이를 기준으로 함), 투약 14일째 및 28일째 결석의 배출 여부 (non-contrast CT, IVU, KUB 등으로 확인하며 KUB는 첫 진단 시 명백한 radio-opaque stone인 경우에만 허용함), 4주 이내에서 결석 배출까지 기간, 진통제를 사용한 횟수와 양, 적극적 치료의 비율 (체외충격파쇄석술, 요관경하요관절석술, 요관부목삽입술을 적극적 치료로 간주함).

1. **예측 부작용 및 사용상의 주의 사항**

1. 예측 부작용

- 본 연구에 사용되는 naftopidil 75mg qd 용법은 유의한 안전성 문제 없이 사용할 수 있게 허가된 용량이며, 일반적으로 이상 반응은 3.28%이며 대부분 경미한 것으로 알려져 있음. 중대한 이상반응은 간기능장애 및 황달 (빈도불명), 실신 및 의식상실 (빈도불명)이 나타나는 경우가 있지만 극히 드물었으며, 본 연구에서는 연구 전 간기능장애 환자는 처음부터 제외 기준임. 또한 주요 이상 반응으로는 현기증 0.95%, 어지러움 0.42%, 저혈압(기립성저혈압 포함) 0.20%, 위부불쾌감 0.20% 이었고, 대부분 일과적이고 경미한 부작용이라 비교적 안전하게 사용할 수 있는 약물임.

2. 병용주의 또는 금기: 다음의 약물을 복용하는 환자의 경우 제외 기준이 되지는 않지만 해당 약물의 사용에 주의 또는 금기가 필요하다.

- 이뇨제 및 혈압강화제를 복용중인 환자는 혈압강하 작용이 증강할 우려가 있으므로 감량하는 등 주의한다.

- PDE5 저해작용을 가진 약제 (실데나필, 바데나필 등)와 이 약의 병용투여시 PDE5 억제제의 혈관 이완성 혈압강하 작용이 증가할 수 있어 시험약을 복용중인 14일간은 PDE5 저해제의 복용을 금지한다.

1. **중지∙탈락 기준**

- 중등도 이상의 이상반응 발생으로 투약의 유지가 어렵거나, 어떠한 이상반응이라도 발생하여 환자가 복약 중단을 요구하면 투약을 중단할 예정임.

- 이상 반응과 상관없이 어떠한 이유에서든 환자가 동의를 철회하면 임상 시험 참여를 중지함.

- 또한 임상시험 진행 중 중대한 이상반응의 발생하거나 임상시험을 계속 진행하는 것에 무리가 있다고 판단되는 경우에는 책임연구자는 심사위원회에 임상시험 중지 요청을 하여야 하고, 심사위원회의 결정에 따라 임상시험을 중지할 수 있음

1. **효과 평가기준, 평가 방법 및 해석 방법(통계분석방법)**

- 1차 평가 변수인 투약 14일째 결석의 배출 여부는 non-contrast CT를 기준으로 평가를 하며, 경우에 따라 IVU, KUB 등을 인정함. 그러나 KUB는 첫 진단 시 명백한 radio-opaque stone인 경우에만 허용함. 해석은 intent-to-treat analysis 원칙으로 chi-square test로 유의한 차이가 있는지 검증함.

- 2차 평가 변수인 28일째 결석 배출 여부는 앞에서와 마찬가지로 평가하며, 결석 배출까지 기간은 4주 이내에서 환자의 배출 목격이나 명백한 배출 증상을 기준으로 평가하며, 환자의 주관적 배출이 불명확한 경우는 영상 검사에서 결석이 보이지 않은 날을 기준으로 함. 이의 해석은 t-test와 log-rank test로 검증함. 4주 이내 진통제 사용량과 적극적 치료의 비율은 각각 t-test와 chi-square test로 차이를 검증함.

1. **부작용을 포함한 안전성의 평가기준, 평가 방법 및 보고 방법**

- 이상반응 발생시 이상 반응 내용 및 중대성 여부 (사망, 생명에 위협, 입원, 장애/불구, 선천성 기형/이상 초래, 중요한 의학적 사건, 기타), 예상 여부 (예, 아니오), 인과관계 (확실함, 거의확실함, 가능함, 가능성적음, 평가곤란, 평가불가, 관련없음)를 평가하여 책임연구자에게 보고함.

- 중대 이상 반응의 경우 즉시 책임연구자에게 보고하고, 책임연구자는 피험자보호센터에 보고함.

- 책임연구자는 이상반응 발생시 피험자보호센터의 일반적인 기준에 준하여 보고함.

1. **자료안전성 모니터링 계획(DSMP)**

- 본 연구는 기 판매되고 있는 약의 허가된 용량을 적용하며 이상반응의 빈도가 높지 않은 약물이므로 저위험 연구에 해당됨.

- 모니터링 책임자: 비뇨기과 전임의 김명

- 모니터링 빈도: 환자를 모집하거나 추적 경과를 보는 기간 동안에는 최소 2주에 1회 이상

- 타기관에서 발생한 이상 반응도 최소 2주에 1회 이상 확인함

- 본 기관 및 타 기관에서 중대 이상 반응이 발생하지 않는 경우는 모니터링 결과를 최종 보고시에 같이 보고함 (안전성 관련 정보 보고서).

- 만약 본 기관 및 타 기관에서 중대 이상 반응이 발생하는 경우 그 내용에 대해 “이상 약물/의료기기 반응 보고서 (본원 또는 본원 외 국내/해외용)”를 피험자보호센터 일반적인 기준에 준하여 보고함

- 본원 및 타 기관에서 사망 또는 생명위협에 해당하는 중대한 이상 반응이 1건이라도 보고되거나, 기타 중대 이상 반응이 거의 확실함 또는 확실함의 인과 관계로 3회 이상 발생시 책임연구자는 공동연구자들과 연구 조기 종료를 상의하고 그 결과를 피험자보호센터에 보고함.

1. **연구대상자의 안전보호를 위한 대책**
2. **연구대상자의 동의 과정**

동의서의 취득은 책임 연구자 또는 책임 연구자로부터 위임을 받은 원내 공동 연구자가 연구대상자에게 직접 받을 예정이며, 연구대상자에게 받을 수 없는 부득이한 특수 경우에만 연구대상자의 법정대리인에게 받을 것입니다. 연구대상자 또는 법정대리인이 동의서 등을 읽을 수 없는 경우에는 공정한 입회자가 동의를 얻는 전 과정에 참석해 피험자 또는 대리인에게 읽어 주고 설명한 후, 피험자 또는 대리인이 피험자의 연구 참여를 구두로 동의하면 가능하다면 동의서 서식에 자필로 서명하고 해달 날짜를 기재하게 한 다음, 공정한 입회자가 동의서 서식에 서명하고 자필로 해당 날짜를 기재하도록 할 것입니다.

모든 동의서 취득 과정에서 연구자는 피험자 또는 대리인에게 강제나 부당한 영향을 미치지 않도록 최대한 객관적인 태도를 취할 것이며, 피험자 또는 대리인이 연구의 모든 정보를 충분히 이해할 수 있는 쉬운 용어 및 언어로 작성된 동의서와 설명을 제공한 후 충분히 생각할 기회와 시간을 주고 자발적으로 동의 의사를 밝힐 때에 동의를 취득할 것입니다. 이때 사용되는 동의서 서식은 연구자가 IRB의 서면승인의 직인이 찍힌 동의서를 사용하며, 피험자 또는 대리인과 동의를 받은 연구자는 동의서 서식에 자필로 서명하고 자필로 해당 날짜를 기재할 것입니다. 피험자 또는 대리인은 연구 과정에서 문의 사항이 있거나 문제가 있을 때 연락할 수 있는 연구자의 연락처와 피험자보호센터의 연락처가 기재된 동의서 1부를 제공받을 것입니다.

또한 피험자의 동의에 영향을 줄 수 있는 새로운 연구 관련 정보가 수집되면 동의서 등 피험자에게 제공될 문서화된 정보는 수정되어 반드시 IRB의 재승인을 받아 사용될 것이며, 피험자의 지속적인 연구 참여 의지에 영향을 줄 경우 연구책임자는 연구대상자 또는 대리인에게 즉시 알리고, 이러한 고지와 관련된 모든 사항을 문서화할 것입니다.

1. **연구대상자의 보상 방안**

약을 추가로 복용하시는 것 이외 모든 검사와 치료의 방법은 통상적인 방법을 유지하기 때문에 추가의 비용이 발생하지 않으며 약은 무료로 제공됩니다. 또한 본 연구는 순수 학술 연구로 별도의 사례비는 없습니다.

임상시험 중 또는 임상시험 이후에도, 연구자는 임상적으로 의미 있는 실험실적 검사치의 이상을 포함하여 임상시험에서 발생한 모든 이상반응에 대해 피험자가 적절한 의학적 처치를 받을 수 있도록 조치하여야 하고, 연구자가 알게 된 피험자의 병발질환에 대해 의학적 처치가 필요한 경우 이를 피험자에게 알려주어야 함. 임상시험으로 인한 피해가 발생하였을 경우 피해자 보상에 대한 규약 (별첨) 에 따라 처리함.

1. **연구대상자의 사생활 보호 및 비밀 보장에 대한 방안**

환자의 사생활 및 개인 신상 정보 보장을 위해 아래와 같은 정보보안 조치를 취할 예정임.

- 연구를 위해 수집되는 정보는 잠금 장치가 있는 연구실에 비밀번호 (password)가 걸린 파일로 보관될 것임.

- 연구 기간 동안 임시로 개인 식별자가 있는 기록이 수집되는 경우에는 연구책임자가 연구를 위해 필요한 경우에만 접근할 것임.

- 자료 수집 후 개인이 식별 가능한 필드들 (이름, 차트번호 등)는 제거할 것임. 개인의 식별이 가능한 필드는 별도 파일에 보관하고 이 파일은 책임연구자가 보관할 것임. 이러한 자료에 대한 접근은 책임연구자 및 그 권한 위임자만이 할 수 있도록 보관됨.

- 연구 결과가 발표되는 경우 개인의 신원을 확인할 수 없는 형태로 발표될 것임.

1. **취약한 연구대상자를 포함하는 경우 추가적인 보호조치 방안**

취약한 연구대상자 (미성년자 / 임산부, 태아, 신생아 / 손상된 동의 능력을 가진 성인 / 학생, 피고용자 / 수감자)는 연구대상으로 하지 않음.

1. **연구의 윤리성 확보를 위한 방안**
2. **규정 및 윤리의 준수**

임상시험관리기준(KGCP): 본 연구를 실시함에 있어 KGCP 및 아래 별도 첨부한 2008년 제 59차 세계의사회 서울총회에서 개정된 Helsinki 선언(아래 참고)의 근본 정신을 준수하여 윤리적이고 과학적인 배려 하에 연구를 실시하도록 한다.

* Helsinki 선언 (2008년 29차 세계의사회 개정안)

A. INTRODUCTION

1. The World Medical Association (WMA) has developed the Declaration of Helsinki as a statement of ethical principles for medical research involving human subjects, including research on identifiable human material and data.

The Declaration is intended to be read as a whole and each of its constituent paragraphs should not be applied without consideration of all other relevant paragraphs.

2. Although the Declaration is addressed primarily to physicians, the WMA encourages other participants in medical research involving human subjects to adopt these principles.

3. It is the duty of the physician to promote and safeguard the health of patients, including those who are involved in medical research. The physician's knowledge and conscience are dedicated to the fulfilment of this duty.

4. The Declaration of Geneva of the WMA binds the physician with the words, "The health of my patient will be my first consideration," and the International Code of Medical Ethics declares that, "A physician shall act in the patient's best interest when providing medical care."

5. Medical progress is based on research that ultimately must include studies involving human subjects. Populations that are underrepresented in medical research should be provided appropriate access to participation in research.

6. In medical research involving human subjects, the well-being of the individual research subject must take precedence over all other interests.

7. The primary purpose of medical research involving human subjects is to understand the causes, development and effects of diseases and improve preventive, diagnostic and therapeutic interventions (methods, procedures and treatments). Even the best current interventions must be evaluated continually through research for their safety, effectiveness, efficiency, accessibility and quality.

8. In medical practice and in medical research, most interventions involve risks and burdens.

9. Medical research is subject to ethical standards that promote respect for all human subjects and protect their health and rights. Some research populations are particularly vulnerable and need special protection. These include those who cannot give or refuse consent for themselves and those who may be vulnerable to coercion or undue influence.

10. Physicians should consider the ethical, legal and regulatory norms and standards for research involving human subjects in their own countries as well as applicable international norms and standards. No national or international ethical, legal or regulatory requirement should reduce or eliminate any of the protections for research subjects set forth in this Declaration.

B. BASIC PRINCIPLES FOR ALL MEDICAL RESEARCH

11. It is the duty of physicians who participate in medical research to protect the life, health, dignity, integrity, right to self-determination, privacy, and confidentiality of personal information of research subjects.

12. Medical research involving human subjects must conform to generally accepted scientific principles, be based on a thorough knowledge of the scientific literature, other relevant sources of information, and adequate laboratory and, as appropriate, animal experimentation. The welfare of animals used for research must be respected.

13. Appropriate caution must be exercised in the conduct of medical research that may harm the environment.

14. The design and performance of each research study involving human subjects must be clearly described in a research protocol. The protocol should contain a statement of the ethical considerations involved and should indicate how the principles in this Declaration have been addressed. The protocol should include information regarding funding, sponsors, institutional affiliations, other potential conflicts of interest, incentives for subjects and provisions for treating and/or compensating subjects who are harmed as a consequence of participation in the research study. The protocol should describe arrangements for post-study access by study subjects to interventions identified as beneficial in the study or access to other appropriate care or benefits.

15. The research protocol must be submitted for consideration, comment, guidance and approval to a research ethics committee before the study begins. This committee must be independent of the researcher, the sponsor and any other undue influence. It must take into consideration the laws and regulations of the country or countries in which the research is to be performed as well as applicable international norms and standards but these must not be allowed to reduce or eliminate any of the protections for research subjects set forth in this Declaration. The committee must have the right to monitor ongoing studies. The researcher must provide monitoring information to the committee, especially information about any serious adverse events. No change to the protocol may be made without consideration and approval by the committee.

16. Medical research involving human subjects must be conducted only by individuals with the appropriate scientific training and qualifications. Research on patients or healthy volunteers requires the supervision of a competent and appropriately qualified physician or other health care professional. The responsibility for the protection of research subjects must always rest with the physician or other health care professional and never the research subjects, even though they have given consent.

17. Medical research involving a disadvantaged or vulnerable population or community is only justified if the research is responsive to the health needs and priorities of this population or community and if there is a reasonable likelihood that this population or community stands to benefit from the results of the research.

18. Every medical research study involving human subjects must be preceded by careful assessment of predictable risks and burdens to the individuals and communities involved in the research in comparison with foreseeable benefits to them and to other individuals or communities affected by the condition under investigation.

19. Every clinical trial must be registered in a publicly accessible database before recruitment of the first subject.

20. Physicians may not participate in a research study involving human subjects unless they are confident that the risks involved have been adequately assessed and can be satisfactorily managed. Physicians must immediately stop a study when the risks are found to outweigh the potential benefits or when there is conclusive proof of positive and beneficial results.

21. Medical research involving human subjects may only be conducted if the importance of the objective outweighs the inherent risks and burdens to the research subjects.

22. Participation by competent individuals as subjects in medical research must be voluntary. Although it may be appropriate to consult family members or community leaders, no competent individual may be enrolled in a research study unless he or she freely agrees.

23. Every precaution must be taken to protect the privacy of research subjects and the confidentiality of their personal information and to minimize the impact of the study on their physical, mental and social integrity.

24. In medical research involving competent human subjects, each potential subject must be adequately informed of the aims, methods, sources of funding, any possible conflicts of interest, institutional affiliations of the researcher, the anticipated benefits and potential risks of the study and the discomfort it may entail, and any other relevant aspects of the study. The potential subject must be informed of the right to refuse to participate in the study or to withdraw consent to participate at any time without reprisal. Special attention should be given to the specific information needs of individual potential subjects as well as to the methods used to deliver the information. After ensuring that the potential subject has understood the information, the physician or another appropriately qualified individual must then seek the potential subject's freely-given informed consent, preferably in writing. If the consent cannot be expressed in writing, the non-written consent must be formally documented and witnessed.

25. For medical research using identifiable human material or data, physicians must normally seek consent for the collection, analysis, storage and/or reuse. There may be situations where consent would be impossible or impractical to obtain for such research or would pose a threat to the validity of the research. In such situations the research may be done only after consideration and approval of a research ethics committee.

26. When seeking informed consent for participation in a research study the physician should be particularly cautious if the potential subject is in a dependent relationship with the physician or may consent under duress. In such situations the informed consent should be sought by an appropriately qualified individual who is completely independent of this relationship.

27. For a potential research subject who is incompetent, the physician must seek informed consent from the legally authorized representative. These individuals must not be included in a research study that has no likelihood of benefit for them unless it is intended to promote the health of the population represented by the potential subject, the research cannot instead be performed with competent persons, and the research entails only minimal risk and minimal burden.

28. When a potential research subject who is deemed incompetent is able to give assent to decisions about participation in research, the physician must seek that assent in addition to the consent of the legally authorized representative. The potential subject's dissent should be respected.

29. Research involving subjects who are physically or mentally incapable of giving consent, for example, unconscious patients, may be done only if the physical or mental condition that prevents giving informed consent is a necessary characteristic of the research population. In such circumstances the physician should seek informed consent from the legally authorized representative. If no such representative is available and if the research cannot be delayed, the study may proceed without informed consent provided that the specific reasons for involving subjects with a condition that renders them unable to give informed consent have been stated in the research protocol and the study has been approved by a research ethics committee. Consent to remain in the research should be obtained as soon as possible from the subject or a legally authorized representative.

30. Authors, editors and publishers all have ethical obligations with regard to the publication of the results of research. Authors have a duty to make publicly available the results of their research on human subjects and are accountable for the completeness and accuracy of their reports. They should adhere to accepted guidelines for ethical reporting. Negative and inconclusive as well as positive results should be published or otherwise made publicly available. Sources of funding, institutional affiliations and conflicts of interest should be declared in the publication. Reports of research not in accordance with the principles of this Declaration should not be accepted for publication.

C. ADDITIONAL PRINCIPLES FOR MEDICAL RESEARCH COMBINED WITH MEDICAL CARE

31. The physician may combine medical research with medical care only to the extent that the research is justified by its potential preventive, diagnostic or therapeutic value and if the physician has good reason to believe that participation in the research study will not adversely affect the health of the patients who serve as research subjects.

32. The benefits, risks, burdens and effectiveness of a new intervention must be tested against those of the best current proven intervention, except in the following circumstances:

 The use of placebo, or no treatment, is acceptable in studies where no current proven intervention exists; or

 Where for compelling and scientifically sound methodological reasons the use of placebo is necessary to determine the efficacy or safety of an intervention and the patients who receive placebo or no treatment will not be subject to any risk of serious or irreversible harm. Extreme care must be taken to avoid abuse of this option.

33. At the conclusion of the study, patients entered into the study are entitled to be informed about the outcome of the study and to share any benefits that result from it, for example, access to interventions identified as beneficial in the study or to other appropriate care or benefits.

34. The physician must fully inform the patient which aspects of the care are related to the research. The refusal of a patient to participate in a study or the patient's decision to withdraw from the study must never interfere with the patient-physician relationship.

35. In the treatment of a patient, where proven interventions do not exist or have been ineffective, the physician, after seeking expert advice, with informed consent from the patient or a legally authorized representative, may use an unproven intervention if in the physician's judgement it offers hope of saving life, re-establishing health or alleviating suffering. Where possible, this intervention should be made the object of research, designed to evaluate its safety and efficacy. In all cases, new information should be recorded and, where appropriate, made publicly available.

1. **참고 문헌**

1. Ramello A, Vitale C, Marangella M. Epidemiology of nephrolithiasis. J Nephrol 2000;13 Suppl 3:S45-50.

2. Stamatelou KK, Francis ME, Jones CA, Nyberg LM, Curhan GC. Time trends in reported prevalence of kidney stones in the United States: 1976-1994. Kidney Int 2003;63:1817-23.

3. Lee HN, Yoon HN, Shim BS. The trend change of incidence and treatment of urolithiasis between the 1980s and 2000s. Korean J Urol 2007;48:40-4.

4. Preminger GM, Tiselius HG, Assimos DG, Alken P, Buck AC, Gallucci M, et al. 2007 Guideline for the management of ureteral calculi. Eur Urol 2007;52:1610-31.

5. Seitz C, Liatsikos E, Porpiglia F, Tiselius HG, Zwergel U. Medical therapy to facilitate the passage of stones: what is the evidence? Eur Urol 2009;56:455-71.

6. Singh A, Alter HJ, Littlepage A. A systematic review of medical therapy to facilitate passage of ureteral calculi. Ann Emerg Med 2007;50:552-63.

7. Zhu Y, Duijvesz D, Rovers MM, Lock TM. alpha-Blockers to assist stone clearance after extracorporeal shock wave lithotripsy: a meta-analysis. BJU Int 2010;106:256-61.

8. Han MC, Park YY, Shim BS. Effect of tamsulosin on the expectant treatment of lower ureteral stones. Korean J Urol 2006;47:708-11.

9. Han MC, Jeong WS, Shim BS. Additive expulsion effect of tamsulosin after shock wave lithotripsy for upper ureteral stones. Korean J Urol 2006;47:813-7.

10. Bak CW, Yoon SJ, Chung H. Effects of an α-blocker and terpene mixture for pain control and spontaneous expulsion of ureter stone. Korean J Urol 2007;48:517-21.

11. Choi NY, Ahn SH, Han JH, Jang IH. The effect of tamsulosin and nifedipine on expulsion of ureteral stones after extracorporeal shock wave lithotripsy. Korean J Urol 2008;49:150-4.

12. Kim TH, Oh SY, Moon YT. The effect of tamsulosin on expulsion of ureteral stones after extracorporeal shock wave lithotripsy. Korean J Urol 2008;49:1100-4.

13. Jadad AR, Moore RA, Carroll D, Jenkinson C, Reynolds DJ, Gavaghan DJ, et al. Assessing the quality of reports of randomized clinical trials: is blinding necessary? Control Clin Trials 1996;17:1-12.

14. Kang DI, Cho WY, Kim TH, Chung JM, Park J, Yoon JH, et al. Effect of tamsulosin 0.2 mg on the short-term treatment of urinary stones: multicenter, prospective, randomized study. Korean J Urol 2009;50:586-90.

15. Morita T, Ando M, Kihara K, Oshima H. Function and distribution of autonomic receptors in canine ureteral smooth muscle. Neurourol Urodyn 1994;13:315-21.

16. Park HK, Choi EY, Jeong BC, Kim HH, Kim BK. Localizations and expressions of alpha-1A, alpha-1B and alpha-1D adrenoceptors in human ureter. Urol Res 2007;35:325-9.

17. Cervenakov I, Fillo J, Mardiak J, Kopecny M, Smirala J, Lepies P. Speedy elimination of ureterolithiasis in lower part of ureters with the alpha 1-blocker--Tamsulosin. Int Urol Nephrol 2002;34:25-9.

18. Sigala S, Dellabella M, Milanese G, Fornari S, Faccoli S, Palazzolo F, et al. Evidence for the presence of alpha1 adrenoceptor subtypes in the human ureter. Neurourol Urodyn 2005;24:142-8.

19. Zheng S, Liu LR, Yuan HC, Wei Q. Tamsulosin as adjunctive treatment after shockwave lithotripsy in patients with upper urinary tract stones: a systematic review and meta-analysis. Scand J Urol Nephrol 2010;44:425-32.

20. Kobayashi M, Naya Y, Kino M, et al. Low dose tamsulosin for stone expulsion after extracorporeal shock wave lithotripsy: efficacy in Japanese male patients with ureteral stone. Int J Urol 2008;15:495-8.

21. Kaneko T, Matsushima H, Morimoto H, Tsuzaka Y, Homma Y. Efficacy of low dose tamsulosin in medical expulsive therapy for ureteral stones in Japanese male patients: a randomized controlled study. Int J Urol 2010;17:462-5.

22. Dellabella M, Milanese G, Muzzonigro G. Medical-expulsive therapy for distal ureterolithiasis: randomized prospective study on role of corticosteroids used in combination with tamsulosin-simplified treatment regimen and health-related quality of life. Urology 2005;66:712-5.

23. Porpiglia F, Vaccino D, Billia M, Renard J, Cracco C, Ghignone G, et al. Corticosteroids and tamsulosin in the medical expulsive therapy for symptomatic distal ureter stones: single drug or association? Eur Urol 2006;50:339-44.

24. Takei R, Ikegaki I, Shibata K, Tsujimoto G, Asano T. Naftopidil, a novel alpha1-adrenoceptor antagonist, displays selective inhibition of canine prostatic pressure and high affinity binding to cloned human alpha1-adrenoceptors. Jpn J Pharmacol 1999;79:447-54.

25. [Shibata K](http://www.ncbi.nlm.nih.gov/pubmed?term=Shibata%20K%5BAuthor%5D&cauthor=true&cauthor_uid=7651358), [Foglar R](http://www.ncbi.nlm.nih.gov/pubmed?term=Foglar%20R%5BAuthor%5D&cauthor=true&cauthor_uid=7651358), [Horie K](http://www.ncbi.nlm.nih.gov/pubmed?term=Horie%20K%5BAuthor%5D&cauthor=true&cauthor_uid=7651358), [Obika K](http://www.ncbi.nlm.nih.gov/pubmed?term=Obika%20K%5BAuthor%5D&cauthor=true&cauthor_uid=7651358), [Sakamoto A](http://www.ncbi.nlm.nih.gov/pubmed?term=Sakamoto%20A%5BAuthor%5D&cauthor=true&cauthor_uid=7651358), [Ogawa S](http://www.ncbi.nlm.nih.gov/pubmed?term=Ogawa%20S%5BAuthor%5D&cauthor=true&cauthor_uid=7651358), [Tsujimoto G](http://www.ncbi.nlm.nih.gov/pubmed?term=Tsujimoto%20G%5BAuthor%5D&cauthor=true&cauthor_uid=7651358). KMD-3213, a novel, potent, alpha 1a-adrenoceptor-selective antagonist: characterization using recombinant human alpha 1-adrenoceptors and native tissues. Mol Pharmacol. 1995;48:250-8.

26. Tsuzaka Y, Matsushima H, Kaneko T, Yamaguchi T, Homma Y. Naftopidil vs silodosin in medical expulsive therapy for ureteral stones: a randomized controlled study in Japanese male patients. Int J Urol. 2011;18:792-5.

27. [Sun X](http://www.ncbi.nlm.nih.gov/pubmed?term=Sun%20X%5BAuthor%5D&cauthor=true&cauthor_uid=19233432), [He L](http://www.ncbi.nlm.nih.gov/pubmed?term=He%20L%5BAuthor%5D&cauthor=true&cauthor_uid=19233432), [Ge W](http://www.ncbi.nlm.nih.gov/pubmed?term=Ge%20W%5BAuthor%5D&cauthor=true&cauthor_uid=19233432), [Lv J](http://www.ncbi.nlm.nih.gov/pubmed?term=Lv%20J%5BAuthor%5D&cauthor=true&cauthor_uid=19233432). Efficacy of selective alpha1D-blocker naftopidil as medical expulsive therapy for distal ureteral stones. [J Urol.](http://www.ncbi.nlm.nih.gov/pubmed/19233432) 2009;181:1716-20

28. Preminger GM, Tiselius HG, Assimos DG, Alken P, Buck C, Gallucci M, Knoll T, Lingeman JE, Nakada SY, Pearle MS, Sarica K, Türk C, Wolf JS Jr; EAU/AUA Nephrolithiasis Guideline Panel. 2007 guideline for the management of ureteral calculi. J Urol. 2007;178:2418-34.

**[별첨1] 연구 설명문 및 동의서**

**[별첨2] 증례기록서**

**[별첨3] 연구대상자 보상규약**

**[별첨4] 책임연구자의 이력**

**[별첨5] 기타 - 책임연구자의 2013년 GCP 이수증 1**

**[별첨6] 기타 - 책임연구자의 2013년 GCP 이수증 2**

**[별첨7] 임상시험자자료집**

**[별첨8] 식약처 승인서**

**[별첨9] 기타 – 분당서울대학교병원 IRB 승인서**

**[별첨10] 기타 – 보라매병원 IRB 승인서**
