# Supplementary material for: Medical expulsive therapy for ureter stone using naftopidil: A multicenter, randomized, double-blind, and placebo-controlled trial
Source: PLoS One. 2017 Apr 21;12(4):e0174962. doi: 10.1371/journal.pone.0174962 (PMC5400235; doi:10.1371/journal.pone.0174962)
Supplement: S3 File — (DOCX) [file pone.0174962.s005.docx]

Medical Expulsive Therapy (MET) for ureter stone by using Naftopidil: Multicenter, randomized, double-blind, placebo controlled study. (SNUBH-URO-2012-03)

Ver. 2.0

Department of Urology, Seoul National University Hospital, Seoul, Korea

Chang Wook Jeong

**Study Summary**

| Title | (Korean) 나프토피딜을 이용한 요관 결석에 대한 내과적 배출 요법: 다기관, 무작위배정, 양쪽눈가림, 위약 대조군 연구 |
| --- | --- |
|  | (English) Medical expulsive therapy for ureter stone using Naftopidil: Multicenter, randomized, double-blind, placebo controlled study. |
| Principal Investigator | Chang Wook Jeong |
| Sponsor | No |

| Purpose | This study is to investigate whether naftopidil is effective or not for the spontaneous passage of ureteral stones with sizes of 3 to 10 mm. |
| --- | --- |
| Study desian | Multicenter, randomized, double-blind, placebo controlled study |
| Study period | IRB ~ 16 Nov 2014 |
| Patients and Methods | When the patients were diagnosed with a ureter stone, the aceclofenac 100 mg or the combined medication of tramadol 37.5 mg and acetaminophen 325 mg was prescribed first. Then the patients were 1:1 randomized to receive either naftopidil 75 mg or placebo. Primary endpoint was the stone passage rates at 14 days after medication.  Enrollment  <Inclusion criteria>  a. ≥ 20 years  b. Patients with a single 3 to 10 mm ureter stone (longest diameter) |
| Sample size | 150 patient, competitive enrollment |
| Study design | Primary Purpose: Treatment  Study Phase: Phase 3  Intervention Model: Parallel Assignment  Number of Arms: 2  Masking: Double Blind (Subject, Caregiver, Investigator, Outcomes Assessor)  Allocation: Randomized  Endpoint Classification: Safety/Efficacy Study  Enrollment: 150 [Anticipated] |
| Outcome Measures | Stone passage rate at 14th day of medication |
| Safety Measures | When adverse events occurs, they are evaluated with respect to the followings: corresponding AE term, whether the event is serious or not(death, life-threatening, in patient hospitalization, incapacity/disability, congenital anomaly/birth defect, important medical event, etc), whether or not the event is expected(yes or no), causality(Certain, probable/likely, possible, unlikely, conditional/unclassified, unassessable/unclassifiable, not related) |

1. **Title**

(Korean) 나프토피딜을 이용한 요관 결석에 대한 내과적 배출 요법: 다기관, 무작위배정, 양쪽눈가림, 위약 대조군 연구.

(English) Medical expulsive therapy for ureter stone using Naftopidil: Multicenter, randomized, double-blind, placebo controlled study.

1. **Institution and address**

(Institution) Department of Urology, Seoul National University Hospital, Seoul, Korea

(Address) 110-744, 서울특별시 종로구 대학로 101

1. **Responsible Party and invesetigators**

**[Responsible Party]**

Principal Investigator: Chang Wook Jeong [cwjeong] M.D, Ph.D.

Affiliation: Seoul National University Hospital

Sponsor: Seoul National University Hospital

Collaborators: Dong-A Pharmaceutical Co., Ltd.

**[Investigators]**

Sung Yong Cho, Juhyun Park, Min Chul Cho (Department of Urology, Seoul Metropolitan Government- Seoul National University Boramae Medical Center, Seoul, Korea)

Woong Na (Department of Urology, National Medical Center, Seoul, Korea)

Sang Wook Lee (Department of Urology, Clinical Research Institute, Kangwon National University Hospital, Kangwon National University School of Medicine, Chunchon, Korea)

Jong Jin Oh, Sangchul Lee (Department of Urology, Seoul National University Bundang Hospital, Seongnam, Korea)

Soyeon Ahn (Medical Research Collaboration Center, Seoul National University Bundang Hospital, Seongnam, Korea)

Chang Wook Jeong (Department of Urology, Seoul National University Hospital, Seoul, Korea)

1. **Sponsor information**

Investigator-initiated study without funding

1. **Objectives and purposes of the study**

**1) Objestives**

Urolithiasis occurs in 5-10% of the world's population. It is one of the main reasons for visits to urologists [1,2]. The goal of stone treatment is to remove all stones with minimal complications. Active removal of ureter stones < 10 mm usually includes drug medication, medical expulsive therapy (MET), shock-wave lithotripsy (SWL), and ureteroscopic lithotripsy (URS) [3]. The MET has received a great deal of research attention over the past 10 years. Data from randomized controlled studies and a meta-analysis involving calcium channel blockers and alpha-adrenergic agents have shown its efficacy [4-6].

There are three subtypes of alpha-adrenergic receptors: 1A, 1B, and 1D. Alpha-1D receptors are most abundant in the human distal ureter. Tamsulosin is a drug used to improve urination in men with an enlarged prostate. Because tamsulosin has alpha-1A and -1D selectivity, it has been believed to be effective for MET. The selectivity of tamsulosin for alpha-1A is 3.3-times higher than for alpha-1D [7,8]. Naftopidil is another alpha-1D adrenergic receptor antagonist, with hhe highest documented selectivity for alpha-1D receptor to date. The selectivity of naftopidil for alpha-1D is 3.1-times higher than that for alpha-1A [9]. Thus, the adrenergic effect for the alpha-1D receptor appear greater for naftopidil than tamsulosin. This implies that naftopidil will display higher efficacy of MET than tamsulosin. However, this has not been studied using a well-designed, randomized, placebo-controlled study. Furthermore, no definite recommendations for MET have been formulated concerning the appropriate maximal stone sizes or follow-up periods for MET.

The authors performed a prospective, multicenter, randomized, double-blind, placebo-controlled study to clarify the effect of naftopidil for MET including the appropriate maximal stone size and follow-up periods..

**2) Purpose of the present study**

This study is to investigate whether naftopidil is effective or not for the spontaneous passage of ureteral stones with sizes of 3 to 10 mm. (AUA/EAU guideline) When the patients were diagnosed with a ureter stone, the aceclofenac 100 mg or the combined medication of tramadol 37.5 mg and acetaminophen 325 mg was prescribed first. Then the patients were 1:1 randomized to receive either naftopidil 75 mg or placebo. Primary endpoint was the stone passage rates at 14 days after medication.

Primary outcome: Stone passage rate at 14th day of medication

Secondary outcome:

a. stone passage rate at 28th day of medication

b. Day of stone passage within 4 weeks of medication

c. amount of analgesics used for 28 days of medication

1. **Components and codes of the test drug**

NFTP75, Naftopidil 75mg, Tab. P.O. qd hs

YURONTP3, URO-2012-03용 Naftopidil 75mg/placebo

1. **Disease related**

Ureter stone

1. **Sample size estimation**

When the patients were diagnosed with a ureter stone, the aceclofenac 100 mg or the combined medication of tramadol 37.5 mg and acetaminophen 325 mg was prescribed first. Then the patients were 1:1 randomized to receive either naftopidil 75 mg or placebo. Primary endpoint was the stone passage rates at 14 days after medication.

1. **Inclusion**
   1. ≥ 20 years
   2. Patients with a single 3 to 10 mm ureter stone (longest diameter)
2. **Exclusion**
   1. Presence of multiple ureter stones
   2. Renal insufficiency (serum Cr > 1.4)
   3. Febrile UTI (fever > 38°, evidence of urinary infection)
   4. Pregnancy or breast feeding
   5. Solitary kidney
   6. Hypersensitivity to Naftopidil
   7. Current use of any alpha-blocker, calcium-channel blocker, corticosteroid (within 4 weeks)
   8. Moderate or severe cardiovascular or cerebrovascular disease
   9. Hepatic dysfunction (>2 x normal LFT)
   10. Significant active medical illness which in the opinion of the investigator would preclude protocol treatment
   11. Genetic disorder such as Galatose intolerance, Lapp Lactase deficiency, Glucose-Galactose malabsorption
3. **Sample size estimation**

We are planning a study of independent cases and controls with 1 control(s) per case. Prior data indicate that the probability of exposure among controls is 0.543. (RR 1.45 [1.34-1.57])(Seitz C et al: "Medical Therapy to facilitate the passage of stones: What is the evidence?" Eur Urol 2009;56:455-71)) If the true probability of exposure among cases is 0.805, we will need to study 65 case patients and 65 control patients to be able to reject the null hypothesis that the exposure rates for case and controls are equal with probability (power) 0.9. The Type I error probability associated with this test of this null hypothesis is 0.05. We will use an uncorrected chi-squared statistic to evaluate this null hypothesis. The drop-out rate was 10%. Finally the number of patients included should be 150 for two groups.

1. **Study period**

1 year after IRB approval (16 Nov 2014)

1. **Study design**
2. **Brief summary**

When the patients were diagnosed with a ureter stone, the aceclofenac 100 mg or the combined medication of tramadol 37.5 mg and acetaminophen 325 mg was prescribed first. Then the patients were 1:1 randomized to receive either naftopidil 75 mg or placebo. Primary endpoint was the stone passage rates at 14 days after medication.

This study is investigator-initiated multicenter research, which is not financially supported by pharmaceuticals or other institutions to conduct completely independent study from financial interest. However, the test drug and the placebo drug which are necessary for carrying out this study will be provided by Dong-A Pharmaceutical Co., Ltd. with no strings attached. Also, this study will not generate additional cost since the protocol of this study has the same test and follow-up methods used in actual clinical practice except the fact that taking the drug is added. Each institution will bear research expenses generally occur.

In addition, this study is approved by KFDA for using Naftopidil in non-benign prostatic hyperplasia patients which is not a KFDA approved indication.(No. 12449, June 4, 2013)

1. **Time table**

If needed, extension of IRB approval will be requested at least one month before the approval expired considering IRB approval is valid for a year.(Total research period: 12 months)

|  | Time table (months) | | | | | | | | | | | | |  | |
| --- | --- | --- | --- | --- | --- | --- | --- | --- | --- | --- | --- | --- | --- | --- | --- |
|  | 1 | 2 | 3 | 4 | 5 | 6 | 7 | 8 | 9 | 10 | 11 | 12 |  | |  |
| Randomization |  |  |  |  |  |  |  |  |  |  |  |  |  | |  |
| Drug prescription |  |  |  |  |  |  |  |  |  |  |  |  |  | |  |
| Follow up |  |  |  |  |  |  |  |  |  |  |  |  |  | |  |
| Data collection |  |  |  |  |  |  |  |  |  |  |  |  |  | |  |
| Data analysis |  |  |  |  |  |  |  |  |  |  |  |  |  | |  |
| Manuscript submission |  |  |  |  |  |  |  |  |  |  |  |  |  | |  |

1. **Choice of Control Group and Randomization**

1. Choice of Control Group: Placebo control

2. Randomization:

- Randomized will be implemented by the independent data manager(Seoul National University Bundang Hospital Medical Research Collaborating Center) which do not directly contact with subjects.

- Allocation Ratio: 1(treatment group) :1(control group)

- Conducting randomization depending on each center after stratified according to the clinical center and generating randomized allocation table

- Data manager implement permuted-block random allocation with varying block sizes for serial numbers using randomization software, and keep the list.

3. Blinding:

- Double-blinding

- Result of randomization will completely not be disclosed to all other investigators, patients and patient guardian, and will be revealed during final analysis, in principle.

- Pharmaceutical company providing the drug (Dong-A ST) will put investigational drugs or placebos for 14 days(14 tab.) into the serial number labeled envelops according to the allocation and seal them. Those envelops will be provided without revealing their contents to other investigators. Even in the pharmaceutical company, only the person who sealing the envelops will be informed of the random table, and the table will be kept closed by a clinical trial representative in the company. The random table can be disclosed after final analysis or in the emergency situation that may effect on subjects.

- General principal investigator will keep random tables of all centers closed while principal investigator or pharmacist in clinical trial pharmacy of each institution will keep that of each institution closed. The random table can be disclosed after final analysis or in the emergency situation that may effect on subjects.

1. **Administration: Dosing of Investigational Drug, Method of Administration, Combination Therapy, Grounds for Choosing Certain Control Drug If Used**

1. Administration, Dosing and Method of Investigational Drug: Naftopidil is circulated in Korean market as 25mg, 50mg and 75mg tablet formulas which were approved by KFDA for benign prostatic hyperplasia. Under 100mg dose, adverse reaction rates was not different depending on dose: 2.6% in 25mg, 2.0% in 50mg and 1.2% in 75mg(Appendix Flivas New Drug Application Documents, P 224). Also, drug dose and drug effect show linear relationship.

In practice, empirically, it is considered that there is no difference on frequency of adverse reaction with respect to drug dose, and equivalent dose of Naftopidil for usual dose of other alpha blockers(tamsulosin 0.2mg, doxazosin 4mg, etc.) is accepted as 75mg. Thus, For benign prostatic hyperplasia patients, which is an indication of Naftopidil, Naftopidil 75mg is being used as standard dose without any other dose escalation. This can be supported by prescription frequency of Naftopidil depending on dose in October, 2012: once for 25mg, 28times for 50mg, 161times for 75mg.

If dose are chosen as 50mg or 25mg at first and it doesn’t show enough efficacy, additional study should be followed using 75mg. Meanwhile, if a study design contains all of the doses, 25mg, 50mg and 75mg, the number of subjects would drastically increase. Also, considering period of drug therapy and the fact that time duration of primary outcome is two weeks, there is a practical difficulty to 1~2 weeks interval dose escalation.

Hence, this study, the first well-designed RCT study that Naftopidil is used for medical expulsive therapy, choose 75mg for test dose since 75mg qd dosage can be maximize plasma concentration resulting in showing maximum effect while safe administration is possible for adult. It is general therapy method to implement more active therapy after monitoring for 2~4 weeks to protect kidney function and to reduce various complications. Therefore, Naftopidil 75mg qd for 14 days dosage is selected.

2. Ground for Choosing Certain Control Drug If Used: Placebo –There has been no placebo-controlled study to prove the effect of naftopidil on medical expulsive therapy for ureteral stone. Thus, placebo is selected as a control. Placebo will be manufactured having the same characteristics such as size, color and weight with naftopidil 75mg, and the same type and amount of excipient will be used except for the active ingredient. More specific information about the component will be provided as the form of component statement or relevant documents when placebo drugs are offered after manufacturing.

3. Combination Therapy: Aceclofenac 100mg, an usual analgesic for ureteral stone, or Ultracet(tramadol 37.5mg/acetaminophen 325mg), when the pain is not controlled by aceclofenac, can be taken for both groups as needed. So, patients are allowed to be treated by basic standard therapy and guaranteed not to feel unnecessary pain.

1. **Observation List, Clinical Examination, and Examination Method**

Age, Gender, location of ureteral stone(left/right), location of stone when first diagnosed(upper/middle/lower segments), longest diameter of ureteral stone(CT result would be considered preferentially when CT was conducted, while KUB or IVP results would be considered when only those tests are conducted), whether ureteral stone pass through or not at 14^th^ and 28^th^ day of medication(It can be observed by using non-contrast CT, IVU or KUB; KUB is allowed to be used only when it is clearly diagnosed radio-opaque stone at first diagnosis), time to stone passage within 4 weeks, the number of use of analgesics and used amount of analgesics, active treatment rate(extracorporeal shock wave lithotripsy, ureteroscopic ureterolithotomy and ureteral stent placement will be considered as active treatment)

1. **Expected Adverse Effect and Warning**

1. Expected Adverse Effect

The dosage used in this study, naftopidil 75mg qd, is approved dose which shows no significant safety issue, and adverse reaction rate is 3.28%, generally, most of those are slight one. Serious adverse reactions such as hepatic dysfunction or jaundice(unknown rate) and fainting or loss of consciousness(unknown rate) were occurred, but very rarely, and hepatic dysfunction is one of exclusion criteria. Also, major adverse reactions were dizziness(0.95%), vertigo(0.42%), hypotension(including orthostatic hypotension, 0.20%) and abdominal discomfort(0.20%), which are mostly transient and slight adverse reaction.

2. Contraindicated or Cautious Drug: Patients who takes the following drugs, although these are not exclusion criteria, should be cautious or contraindicated when take those drugs.

- Patients who takes diuretics or antihypertensive drug should be cautious or reduce the dose due to possible enhancement of antihypertensive effect.

- Coadministration of PDE5 inhibited drugs(sildenafil, vardenafil, etc.) and this drug can increase vasodilational hypotensive effect. Hence, PDE5 inhibitors are not allowed for 14days during test drugs are administrated.

1. **Criteria for Discontinuation and Exclusion**

- Administration will be discontinued when it is hard to continue the administration due to moderate or severe adverse reaction or when patients ask for discontinue participation due to any adverse reaction.

- At any time, if a patient withdraws the consent to participate the study, regardless of any adverse reaction, the patient will be excluded in the study.

- In addition, when any serious adverse reaction occurs or it seems to be hard to continue clinical trials, principal investigator should ask discontinuation of the clinical trials to IRB. And the clinical trials will be discontinued depending on the decision of IRB.

1. **Criteria for Efficacy Evaluation, Evaluation Methods and Interpretation Method(Statistical Method)**

- Primary endpoint, whether or not stone pass through at 14^th^ day of medication, will be evaluated based on non-contrast CT, while, by cases, IVU or KUB can be allowed to be used. However, KUB will be only allowed when it is clearly concluded as radio-opaque stone case in the first diagnosis. Interpretation will be carried out by intent-to-treat analysis, in principle, and identifying statistical significant differences using chi-square test.

- Secondary endpoint, whether or not stone pass through at 28^th^ day of medication, will be evaluated using the same method with primary endpoint. Time to stone passage will be determined as the day when a patients observe the stone passage or there is an obvious passage symptom within 4 weeks, while imaging examination data may be used if subjective judgement by a patient is not clear. Interpretation will be conducted by t-test and log-rank test. Amount of analgesics used for 4 weeks and rate of active treatment will be analyzed by using t-test and chi-square test, respectively.

1. **Criteria for Safety Evaluation including Side Effect, Evaluation Methods and Reporting Methods**

- When adverse events occurs, they are evaluated and reported to PI. The evaluation criteria is as following: corresponding AE term, whether the event is serious or not(death, life-threatening, in patient hospitalization, incapacity/disability, congenital anomaly/birth defect, important medical event, etc), whether or not the event is expected(yes or no), causality(Certain, probable/likely, possible, unlikely, conditional/unclassified, unassessable/unclassifiable, not related)

- When serious adverse reactions occur, it should be immediately reported to IP. Then PI should report them to Human Research Protection Center.

-.PI report the details to Human Research Protection Center based on general criteria when adverse reactions occurs.

1. **Data Safety Monitoring Plan(DSMP)**

- This study belongs to low risk research since already approved drug and dosage will be used, which has also low frequency of adverse reaction.

- Person in Charge of Monitoring: Kim Myung, Fellow in Urology

- Frequency of Monitoring: At least once every other week during patients recruiting period or follow up period

- Adverse reactions occurred in other center also should be checked at least once every other week.

- If no serious adverse reaction occurs in any center including this center, the results of monitoring will be reported in the final report(Safety Related Information Report).

- If serious adverse reactions occur in this or any other center, it should be reported as a form of “Adverse Drug/Medical Device reaction Report(This center or other domestic/oversee centers) to Human Research Protection Center based on general criteria.

- If death or life-threatening serious adverse reactions occur at least once in any center or any other serious adverse reaction occur 3 or more times with certain or probable/likely casualty, PI and co-investigators should discuss early termination of the clinical trials and the result should be reported to Human Research Protection Center.

1. **Measures to Protect Human Subject**
2. **Process of Informed Consent**

PI or authorized co-investigators in the center will obtain an informed consent directly from a subject, and only when it is unavailable, the informed consent will be obtained from a legal representative of a subject. If a subject or a legal representative of a subject can’t read the informed consent, an impartial third party will verbally read and explain it to the subject or the legal representative. If the subject of legal representative verbally consents to participate in the research, he/she will be guided to sign the informed consent form and fill in the date while the third party will also sign the consent form and fill in the date.

All the process obtaining a consent will be implemented in the most objective manner to avoid force or improper influence. The consent will be obtained when the subject present their consent voluntarily after enough time to consider as well as provided information about the study and the consent form written in basic and straightforward vocabulary. The informed consent form containing IRB approval stamp will be used, and an investigator will sign the consent form and fill in the date after obtaining consent from a subject or representative. A subject or legal representative will be provided a copy of informed consent form containing contact information of investigators and Human Research Protection Center for any inquiry during the study.

Also, when new information that can effect on consent of subjects is collected, the information documented for providing to subjects must be updated and re-approval by IRB. If the new information affects a subject’s opinion whether to continue participation, PI should promptly notify to a subject or representative and all the relevant notification should be documented.

1. **Compensation for Subjects**

Since all examination or treatment will follow standard methods except additionally taking the drug, no additional cost will be generated and the drug will be provided for free. Also, there is no pay rewards for this study due to its pure academic purpose

During clinical trials or after the trials, investigators should take proper measures to treat adverse reaction occurred on subjects including abnormal lab test values that is scientifically meaningful. All the intercurrent disease that investigator become to notice should be informed to a subject if medical measures is needed. If any injury occurs due to the clinical trials, it should be dealt with according to Compensation for Injury(appendix).

1. **Protecting the Privacy of Subjects and Confidentiality**

For protecting the privacy of subjects, the following security measures will be taken

- Data collected for a research will be kept in password-protected files at locked research office.

- When records with individual identifier are collected, temporally, it will be assessed only if needed by PI.

- After collecting data, fields that can identify each individual, such as name or chart No, will be eliminated. The fields identifiable for each individual will be stored in separate files and hold by PI.

- If the study results will be released, the result will be shown in the form that is not available to distinguish each individual.

1. **Additional Protection Measures for Vulnerable Subjects**

This study will not recruite vulnerable subjects(children/pregnant women, fetus, neoate/cognitively impaired person/students,employees/prisoners)

1. **Measures for Ethical Concerns**
2. **Conformity to Regulation and Ethics**

Korean Good Clinical Practice(KGCP): This study will be conducted in an ethical, and scientifically considered way, along with conforming to KGCP and Declaration of Helsinki attached as below(amended by the 59^th^ WMA General Assembly, Seoul, South Korea, Oct. 2008)

* Helsinki Declaration

A. INTRODUCTION

1. The World Medical Association (WMA) has developed the Declaration of Helsinki as a statement of ethical principles for medical research involving human subjects, including research on identifiable human material and data.

The Declaration is intended to be read as a whole and each of its constituent paragraphs should not be applied without consideration of all other relevant paragraphs.

2. Although the Declaration is addressed primarily to physicians, the WMA encourages other participants in medical research involving human subjects to adopt these principles.

3. It is the duty of the physician to promote and safeguard the health of patients, including those who are involved in medical research. The physician's knowledge and conscience are dedicated to the fulfilment of this duty.

4. The Declaration of Geneva of the WMA binds the physician with the words, "The health of my patient will be my first consideration," and the International Code of Medical Ethics declares that, "A physician shall act in the patient's best interest when providing medical care."

5. Medical progress is based on research that ultimately must include studies involving human subjects. Populations that are underrepresented in medical research should be provided appropriate access to participation in research.

6. In medical research involving human subjects, the well-being of the individual research subject must take precedence over all other interests.

7. The primary purpose of medical research involving human subjects is to understand the causes, development and effects of diseases and improve preventive, diagnostic and therapeutic interventions (methods, procedures and treatments). Even the best current interventions must be evaluated continually through research for their safety, effectiveness, efficiency, accessibility and quality.

8. In medical practice and in medical research, most interventions involve risks and burdens.

9. Medical research is subject to ethical standards that promote respect for all human subjects and protect their health and rights. Some research populations are particularly vulnerable and need special protection. These include those who cannot give or refuse consent for themselves and those who may be vulnerable to coercion or undue influence.

10. Physicians should consider the ethical, legal and regulatory norms and standards for research involving human subjects in their own countries as well as applicable international norms and standards. No national or international ethical, legal or regulatory requirement should reduce or eliminate any of the protections for research subjects set forth in this Declaration.

B. BASIC PRINCIPLES FOR ALL MEDICAL RESEARCH

11. It is the duty of physicians who participate in medical research to protect the life, health, dignity, integrity, right to self-determination, privacy, and confidentiality of personal information of research subjects.

12. Medical research involving human subjects must conform to generally accepted scientific principles, be based on a thorough knowledge of the scientific literature, other relevant sources of information, and adequate laboratory and, as appropriate, animal experimentation. The welfare of animals used for research must be respected.

13. Appropriate caution must be exercised in the conduct of medical research that may harm the environment.

14. The design and performance of each research study involving human subjects must be clearly described in a research protocol. The protocol should contain a statement of the ethical considerations involved and should indicate how the principles in this Declaration have been addressed. The protocol should include information regarding funding, sponsors, institutional affiliations, other potential conflicts of interest, incentives for subjects and provisions for treating and/or compensating subjects who are harmed as a consequence of participation in the research study. The protocol should describe arrangements for post-study access by study subjects to interventions identified as beneficial in the study or access to other appropriate care or benefits.

15. The research protocol must be submitted for consideration, comment, guidance and approval to a research ethics committee before the study begins. This committee must be independent of the researcher, the sponsor and any other undue influence. It must take into consideration the laws and regulations of the country or countries in which the research is to be performed as well as applicable international norms and standards but these must not be allowed to reduce or eliminate any of the protections for research subjects set forth in this Declaration. The committee must have the right to monitor ongoing studies. The researcher must provide monitoring information to the committee, especially information about any serious adverse events. No change to the protocol may be made without consideration and approval by the committee.

16. Medical research involving human subjects must be conducted only by individuals with the appropriate scientific training and qualifications. Research on patients or healthy volunteers requires the supervision of a competent and appropriately qualified physician or other health care professional. The responsibility for the protection of research subjects must always rest with the physician or other health care professional and never the research subjects, even though they have given consent.

17. Medical research involving a disadvantaged or vulnerable population or community is only justified if the research is responsive to the health needs and priorities of this population or community and if there is a reasonable likelihood that this population or community stands to benefit from the results of the research.

18. Every medical research study involving human subjects must be preceded by careful assessment of predictable risks and burdens to the individuals and communities involved in the research in comparison with foreseeable benefits to them and to other individuals or communities affected by the condition under investigation.

19. Every clinical trial must be registered in a publicly accessible database before recruitment of the first subject.

20. Physicians may not participate in a research study involving human subjects unless they are confident that the risks involved have been adequately assessed and can be satisfactorily managed. Physicians must immediately stop a study when the risks are found to outweigh the potential benefits or when there is conclusive proof of positive and beneficial results.

21. Medical research involving human subjects may only be conducted if the importance of the objective outweighs the inherent risks and burdens to the research subjects.

22. Participation by competent individuals as subjects in medical research must be voluntary. Although it may be appropriate to consult family members or community leaders, no competent individual may be enrolled in a research study unless he or she freely agrees.

23. Every precaution must be taken to protect the privacy of research subjects and the confidentiality of their personal information and to minimize the impact of the study on their physical, mental and social integrity.

24. In medical research involving competent human subjects, each potential subject must be adequately informed of the aims, methods, sources of funding, any possible conflicts of interest, institutional affiliations of the researcher, the anticipated benefits and potential risks of the study and the discomfort it may entail, and any other relevant aspects of the study. The potential subject must be informed of the right to refuse to participate in the study or to withdraw consent to participate at any time without reprisal. Special attention should be given to the specific information needs of individual potential subjects as well as to the methods used to deliver the information. After ensuring that the potential subject has understood the information, the physician or another appropriately qualified individual must then seek the potential subject's freely-given informed consent, preferably in writing. If the consent cannot be expressed in writing, the non-written consent must be formally documented and witnessed.

25. For medical research using identifiable human material or data, physicians must normally seek consent for the collection, analysis, storage and/or reuse. There may be situations where consent would be impossible or impractical to obtain for such research or would pose a threat to the validity of the research. In such situations the research may be done only after consideration and approval of a research ethics committee.

26. When seeking informed consent for participation in a research study the physician should be particularly cautious if the potential subject is in a dependent relationship with the physician or may consent under duress. In such situations the informed consent should be sought by an appropriately qualified individual who is completely independent of this relationship.

27. For a potential research subject who is incompetent, the physician must seek informed consent from the legally authorized representative. These individuals must not be included in a research study that has no likelihood of benefit for them unless it is intended to promote the health of the population represented by the potential subject, the research cannot instead be performed with competent persons, and the research entails only minimal risk and minimal burden.

28. When a potential research subject who is deemed incompetent is able to give assent to decisions about participation in research, the physician must seek that assent in addition to the consent of the legally authorized representative. The potential subject's dissent should be respected.

29. Research involving subjects who are physically or mentally incapable of giving consent, for example, unconscious patients, may be done only if the physical or mental condition that prevents giving informed consent is a necessary characteristic of the research population. In such circumstances the physician should seek informed consent from the legally authorized representative. If no such representative is available and if the research cannot be delayed, the study may proceed without informed consent provided that the specific reasons for involving subjects with a condition that renders them unable to give informed consent have been stated in the research protocol and the study has been approved by a research ethics committee. Consent to remain in the research should be obtained as soon as possible from the subject or a legally authorized representative.

30. Authors, editors and publishers all have ethical obligations with regard to the publication of the results of research. Authors have a duty to make publicly available the results of their research on human subjects and are accountable for the completeness and accuracy of their reports. They should adhere to accepted guidelines for ethical reporting. Negative and inconclusive as well as positive results should be published or otherwise made publicly available. Sources of funding, institutional affiliations and conflicts of interest should be declared in the publication. Reports of research not in accordance with the principles of this Declaration should not be accepted for publication.

C. ADDITIONAL PRINCIPLES FOR MEDICAL RESEARCH COMBINED WITH MEDICAL CARE

31. The physician may combine medical research with medical care only to the extent that the research is justified by its potential preventive, diagnostic or therapeutic value and if the physician has good reason to believe that participation in the research study will not adversely affect the health of the patients who serve as research subjects.

32. The benefits, risks, burdens and effectiveness of a new intervention must be tested against those of the best current proven intervention, except in the following circumstances:

 The use of placebo, or no treatment, is acceptable in studies where no current proven intervention exists; or

 Where for compelling and scientifically sound methodological reasons the use of placebo is necessary to determine the efficacy or safety of an intervention and the patients who receive placebo or no treatment will not be subject to any risk of serious or irreversible harm. Extreme care must be taken to avoid abuse of this option.

33. At the conclusion of the study, patients entered into the study are entitled to be informed about the outcome of the study and to share any benefits that result from it, for example, access to interventions identified as beneficial in the study or to other appropriate care or benefits.

34. The physician must fully inform the patient which aspects of the care are related to the research. The refusal of a patient to participate in a study or the patient's decision to withdraw from the study must never interfere with the patient-physician relationship.

35. In the treatment of a patient, where proven interventions do not exist or have been ineffective, the physician, after seeking expert advice, with informed consent from the patient or a legally authorized representative, may use an unproven intervention if in the physician's judgement it offers hope of saving life, re-establishing health or alleviating suffering. Where possible, this intervention should be made the object of research, designed to evaluate its safety and efficacy. In all cases, new information should be recorded and, where appropriate, made publicly available.

1. **References**

1. Ramello A, Vitale C, Marangella M. Epidemiology of nephrolithiasis. J Nephrol 2000;13 Suppl 3:S45-50.

2. Stamatelou KK, Francis ME, Jones CA, Nyberg LM, Curhan GC. Time trends in reported prevalence of kidney stones in the United States: 1976-1994. Kidney Int 2003;63:1817-23.

3. Lee HN, Yoon HN, Shim BS. The trend change of incidence and treatment of urolithiasis between the 1980s and 2000s. Korean J Urol 2007;48:40-4.

4. Preminger GM, Tiselius HG, Assimos DG, Alken P, Buck AC, Gallucci M, et al. 2007 Guideline for the management of ureteral calculi. Eur Urol 2007;52:1610-31.

5. Seitz C, Liatsikos E, Porpiglia F, Tiselius HG, Zwergel U. Medical therapy to facilitate the passage of stones: what is the evidence? Eur Urol 2009;56:455-71.

6. Singh A, Alter HJ, Littlepage A. A systematic review of medical therapy to facilitate passage of ureteral calculi. Ann Emerg Med 2007;50:552-63.

7. Zhu Y, Duijvesz D, Rovers MM, Lock TM. alpha-Blockers to assist stone clearance after extracorporeal shock wave lithotripsy: a meta-analysis. BJU Int 2010;106:256-61.

8. Han MC, Park YY, Shim BS. Effect of tamsulosin on the expectant treatment of lower ureteral stones. Korean J Urol 2006;47:708-11.

9. Han MC, Jeong WS, Shim BS. Additive expulsion effect of tamsulosin after shock wave lithotripsy for upper ureteral stones. Korean J Urol 2006;47:813-7.

10. Bak CW, Yoon SJ, Chung H. Effects of an α-blocker and terpene mixture for pain control and spontaneous expulsion of ureter stone. Korean J Urol 2007;48:517-21.

11. Choi NY, Ahn SH, Han JH, Jang IH. The effect of tamsulosin and nifedipine on expulsion of ureteral stones after extracorporeal shock wave lithotripsy. Korean J Urol 2008;49:150-4.

12. Kim TH, Oh SY, Moon YT. The effect of tamsulosin on expulsion of ureteral stones after extracorporeal shock wave lithotripsy. Korean J Urol 2008;49:1100-4.

13. Jadad AR, Moore RA, Carroll D, Jenkinson C, Reynolds DJ, Gavaghan DJ, et al. Assessing the quality of reports of randomized clinical trials: is blinding necessary? Control Clin Trials 1996;17:1-12.

14. Kang DI, Cho WY, Kim TH, Chung JM, Park J, Yoon JH, et al. Effect of tamsulosin 0.2 mg on the short-term treatment of urinary stones: multicenter, prospective, randomized study. Korean J Urol 2009;50:586-90.

15. Morita T, Ando M, Kihara K, Oshima H. Function and distribution of autonomic receptors in canine ureteral smooth muscle. Neurourol Urodyn 1994;13:315-21.

16. Park HK, Choi EY, Jeong BC, Kim HH, Kim BK. Localizations and expressions of alpha-1A, alpha-1B and alpha-1D adrenoceptors in human ureter. Urol Res 2007;35:325-9.

17. Cervenakov I, Fillo J, Mardiak J, Kopecny M, Smirala J, Lepies P. Speedy elimination of ureterolithiasis in lower part of ureters with the alpha 1-blocker--Tamsulosin. Int Urol Nephrol 2002;34:25-9.

18. Sigala S, Dellabella M, Milanese G, Fornari S, Faccoli S, Palazzolo F, et al. Evidence for the presence of alpha1 adrenoceptor subtypes in the human ureter. Neurourol Urodyn 2005;24:142-8.

19. Zheng S, Liu LR, Yuan HC, Wei Q. Tamsulosin as adjunctive treatment after shockwave lithotripsy in patients with upper urinary tract stones: a systematic review and meta-analysis. Scand J Urol Nephrol 2010;44:425-32.

20. Kobayashi M, Naya Y, Kino M, et al. Low dose tamsulosin for stone expulsion after extracorporeal shock wave lithotripsy: efficacy in Japanese male patients with ureteral stone. Int J Urol 2008;15:495-8.

21. Kaneko T, Matsushima H, Morimoto H, Tsuzaka Y, Homma Y. Efficacy of low dose tamsulosin in medical expulsive therapy for ureteral stones in Japanese male patients: a randomized controlled study. Int J Urol 2010;17:462-5.

22. Dellabella M, Milanese G, Muzzonigro G. Medical-expulsive therapy for distal ureterolithiasis: randomized prospective study on role of corticosteroids used in combination with tamsulosin-simplified treatment regimen and health-related quality of life. Urology 2005;66:712-5.

23. Porpiglia F, Vaccino D, Billia M, Renard J, Cracco C, Ghignone G, et al. Corticosteroids and tamsulosin in the medical expulsive therapy for symptomatic distal ureter stones: single drug or association? Eur Urol 2006;50:339-44.

24. Takei R, Ikegaki I, Shibata K, Tsujimoto G, Asano T. Naftopidil, a novel alpha1-adrenoceptor antagonist, displays selective inhibition of canine prostatic pressure and high affinity binding to cloned human alpha1-adrenoceptors. Jpn J Pharmacol 1999;79:447-54.

25. [Shibata K](http://www.ncbi.nlm.nih.gov/pubmed?term=Shibata%20K%5BAuthor%5D&cauthor=true&cauthor_uid=7651358), [Foglar R](http://www.ncbi.nlm.nih.gov/pubmed?term=Foglar%20R%5BAuthor%5D&cauthor=true&cauthor_uid=7651358), [Horie K](http://www.ncbi.nlm.nih.gov/pubmed?term=Horie%20K%5BAuthor%5D&cauthor=true&cauthor_uid=7651358), [Obika K](http://www.ncbi.nlm.nih.gov/pubmed?term=Obika%20K%5BAuthor%5D&cauthor=true&cauthor_uid=7651358), [Sakamoto A](http://www.ncbi.nlm.nih.gov/pubmed?term=Sakamoto%20A%5BAuthor%5D&cauthor=true&cauthor_uid=7651358), [Ogawa S](http://www.ncbi.nlm.nih.gov/pubmed?term=Ogawa%20S%5BAuthor%5D&cauthor=true&cauthor_uid=7651358), [Tsujimoto G](http://www.ncbi.nlm.nih.gov/pubmed?term=Tsujimoto%20G%5BAuthor%5D&cauthor=true&cauthor_uid=7651358). KMD-3213, a novel, potent, alpha 1a-adrenoceptor-selective antagonist: characterization using recombinant human alpha 1-adrenoceptors and native tissues. Mol Pharmacol. 1995;48:250-8.

26. Tsuzaka Y, Matsushima H, Kaneko T, Yamaguchi T, Homma Y. Naftopidil vs silodosin in medical expulsive therapy for ureteral stones: a randomized controlled study in Japanese male patients. Int J Urol. 2011;18:792-5.

27. [Sun X](http://www.ncbi.nlm.nih.gov/pubmed?term=Sun%20X%5BAuthor%5D&cauthor=true&cauthor_uid=19233432), [He L](http://www.ncbi.nlm.nih.gov/pubmed?term=He%20L%5BAuthor%5D&cauthor=true&cauthor_uid=19233432), [Ge W](http://www.ncbi.nlm.nih.gov/pubmed?term=Ge%20W%5BAuthor%5D&cauthor=true&cauthor_uid=19233432), [Lv J](http://www.ncbi.nlm.nih.gov/pubmed?term=Lv%20J%5BAuthor%5D&cauthor=true&cauthor_uid=19233432). Efficacy of selective alpha1D-blocker naftopidil as medical expulsive therapy for distal ureteral stones. [J Urol.](http://www.ncbi.nlm.nih.gov/pubmed/19233432) 2009;181:1716-20

28. Preminger GM, Tiselius HG, Assimos DG, Alken P, Buck C, Gallucci M, Knoll T, Lingeman JE, Nakada SY, Pearle MS, Sarica K, Türk C, Wolf JS Jr; EAU/AUA Nephrolithiasis Guideline Panel. 2007 guideline for the management of ureteral calculi. J Urol. 2007;178:2418-34.
